# Gene Coverage Count and Classification (GC_3_): a locus sequence coverage assessment tool using short-read whole genome sequencing data, and its application to identify and classify histidine-rich protein 2 and 3 deletions in *Plasmodium falciparum*

**DOI:** 10.1186/s12936-022-04376-3

**Published:** 2022-11-29

**Authors:** Thomas C. Stabler, Ankit Dwivedi, Biraj Shrestha, Sudhaunshu Joshi, Tobias Schindler, Amed Ouattara, Guillermo A. García, Claudia Daubenberger, Joana C. Silva

**Affiliations:** 1 Department of Medical Parasitology and Infection Biology, Swiss Tropical and Public Health Institute, Basel, Switzerland; 2 University of Basel, Basel, Switzerland; 3 Institute for Genome Sciences, University of Maryland School of Medicine, Baltimore, MD USA; 4 Malaria Research Program, Center for Vaccine Development and Global Health, University of Maryland School of Medicine, Baltimore, MD USA; 5 Medical Care Development International, Silver Spring, MD USA; 6 Department of Microbiology and Immunology, University of Maryland School of Medicine, Baltimore, MD USA

**Keywords:** Malaria, Rapid Diagnostic Test, hrp2, hrp3, Deletion, Gene coverage, Genomics, Bioinformatics

## Abstract

**Background:**

The ability of malaria rapid diagnostic tests (RDTs) to effectively detect active infections is being compromised by the presence of malaria strains with genomic deletions at the *hrp2* and *hrp3* loci, encoding the antigens most commonly targeted in diagnostics for *Plasmodium falciparum* detection. The presence of such deletions can be determined in publically available *P. falciparum* whole genome sequencing (WGS) datasets. A computational approach was developed and validated, termed Gene Coverage Count and Classification (GC_3_), to analyse genome-wide sequence coverage data and provide informative outputs to assess presence and coverage profile of a target locus in WGS data. GC_3_ was applied to detect deletions at *hrp2* and *hrp3* (*hrp2/*3) and flanking genes in different geographic regions and across time points.

**Methods:**

GC_3_ uses Python and R scripts to extract locus read coverage metrics from mapped WGS data according to user-defined parameters and generates relevant tables and figures. GC_3_ was tested using WGS data for laboratory reference strains with known *hrp2/3* genotypes, and its results compared to those of a *hrp2/3*-specific qPCR assay. Samples with at least 25% of coding region positions with zero coverage were classified as having a deletion. Publicly available sequence data was analysed and compared with published deletion frequency estimates.

**Results:**

GC_3_ results matched the expected coverage of known laboratory reference strains. Agreement between GC_3_ and a *hrp2/3*-specific qPCR assay reported for 19/19 (100%) *hrp2* deletions and 18/19 (94.7%) *hrp3* deletions. Among Cambodian (n = 127) and Brazilian (n = 20) WGS datasets, which had not been previously analysed for *hrp2/3* deletions, GC_3_ identified *hrp2* deletions in three and four samples, and *hrp3* deletions in 10 and 15 samples, respectively. Plots of *hrp2/3* coding regions, grouped by year of sample collection, showed a decrease in median standardized coverage among Malawian samples (n = 150) suggesting the importance of a careful, properly controlled follow up to determine if an increase in frequency of deletions has occurred between 2007–2008 and 2014–2015. Among Malian (n = 90) samples, median standardized coverage was lower in 2002 than 2010, indicating widespread deletions present at the gene locus in 2002.

**Conclusions:**

The GC_3_ tool accurately classified *hrp2/3* deletions and provided informative tables and figures to analyse targeted gene coverage. GC_3_ is an appropriate tool when performing preliminary and exploratory assessment of locus coverage data.

**Supplementary Information:**

The online version contains supplementary material available at 10.1186/s12936-022-04376-3.

## Background

From 2010 to 2020, national malaria control programmes (NMCPs) distributed 2.2 billion rapid diagnostic tests (RDTs) for malaria and 3.1 billion RDTs were sold by manufacturers, the majority of these going to malaria-endemic countries in sub-Saharan Africa [1]. RDTs are an integral part of nearly all NMCP’s clinical and field interventions since they provide quick and effective malaria diagnosis. These RDTs include a small cassette detecting *Plasmodium*-specific antigens in the blood of an infected individual and are user-friendly and affordable [2]. Predominantly, RDTs detect the *Plasmodium falciparum*-specific antigen histidine-rich protein 2 (HRP2), which is released into the bloodstream in large quantities when infected red blood cells lyse [3]. *Plasmodium falciparum* accounts for vast majority of the 241 million reported human malaria cases in 2020 and is the primary parasite causing malaria-related mortality and morbidity [1]. Due to considerable sequence similarity between the two proteins, (HRP3 is a truncated protein of HRP2 [4] and the two are encoded by similar loci), HRP3 can also bind to the monoclonal antibody on HRP2-based RDTs, but becomes more apparent in high-density infections [5]. As evidence of their effectiveness, 94% of WHO-qualified RDTs are either HRP2-based or based on a combination of HRP2 and a partner antigen, such as parasite lactate dehydrogenase or aldolase [6–8]. HRP2-based RDTs are an essential diagnostic tool for NMCPs to scale surveillance operations and adequately assess infection, leading to proper treatment administration, measure intervention progress and identify malaria reservoirs.

Recently, however, the effectiveness of HRP2-based RDTs is becoming compromised due to the emergence of deletions in the *hrp2* and *hrp3* (*hrp2/3*) loci that prevent the expression of a detectable protein [6, 8–16]. In particular, full deletions, as well as some partial deletions, in one or both of these genes eliminate HRP2 and/or HRP3 signal on RDTs, preventing accurate malaria diagnosis. Previous estimates of *hrp2/3* deletion prevalence report higher frequencies in South and Central America, followed by Africa, then Asia and Oceania [17]. Low-transmission areas with high treatment rates, characteristics often found in elimination settings, are especially at risk for the spread of strains with *hrp2/3* gene deletions, as models show that, under those conditions, strains with *hrp2/3* deletions have a strong fitness advantage over those with intact genes [18]. Therefore, as NMCPs continue to control and move toward elimination, it is critical to monitor the presence and spread of *hrp2/3* deletions. Without fully understanding the dynamics of *hrp2/3* deletions, and spread of those deletions in particular, undiagnosed infections may lead to an increase in malaria prevalence and mortality, and hinder global progress towards control and elimination.

A computational tool that facilitates detection and classification of deletions in *hrp2/3* (e.g. partial *vs*. complete deletions) in published whole genome sequencing (WGS) datasets will enable rapid and detailed analysis of deletions within datasets, and comparisons between datasets. The development of baseline values as well as the comparison of deletion prevalences across current samples sets as well as temporal comparison between these and previously published datasets may be particularly informative. Previous studies have performed analyses using WGS data [19–21]; however, implementation of the methods used in these studies requires a strong understanding of bioinformatics tools and packages. The development of a more user-friendly computational tool would expand the ability to assess the presence of locus deletions based on WGS coverage data to a wider audience investigating copy number variations, including deletions, in *hrp2/3* or other target genes. This work aimed to fill this gap, by developing a computational tool, termed “Gene Coverage Count and Classification”, or GC_3_, to provide translatable results on the presence of *hrp2/3* deletions and their classification, based on short-read WGS data, among global *P. falciparum* samples for which WGS data is available.

## Methods

### Samples

The WGS data used were generated either by direct sequencing of total DNA extracted from each isolate or by sequencing post selective whole genome amplification (sWGA) of extracted DNA, and were reported previously [22]. Some of the WGS datasets were generated as part of the MalariaGEN project [23] and downloaded from the Sequence Read Archive (SRA). A selection of field samples representing 19 different countries from Africa (n = 9), South America (n = 5), Asia (n = 4) and Oceania (n = 1), for a total of 1120 datasets (1114 global samples + 6 reference strains), were evaluated for general results (Additional file 1: Figure S1). The following laboratory reference strains with known *hrp2* and *hpr3* genotype were used for developing and testing GC_3_: NF54 (West Africa) – *hrp2* and *hrp3* present, 7G8 (Brazil) – *hrp2* and *hrp3* present, NF135.C10 (Cambodia) – *hrp2* and *hrp3* present, NF166 (Guinea) – *hrp2* and *hrp3* present, Dd2 (Laos) – *hrp2* absent/*hrp3* present and HB3 (Honduras) – *hrp2* present/*hrp3* absent.

Read coverage files were generated by aligning raw reads in fastq format to the *Pf*3D7 reference genome assembly (PlasmoDB release v24) using bowtie2 (v2.2.9 and above). Alignment files in BAM (Binary sequence Alignment/Map) format were processed according to GATK’s (Genome Analysis Toolkit) Best Practices documentation. Genome-wide coverage per site was recovered using bedtools’ genomecov function [22, 24]. The resulting BED (Browser Extensible Data) file (a tab-delimited text file) is used as the initial input to GC_3_. However, any delimited file with columns for molecule identifier (e.g. Pf3D7_08_v3), chromosomal position and coverage value is acceptable. When comparing sample datasets, coverage values per base pair (bp) were standardized by dividing ‘locus coverage’ by ‘subtelomeric mean coverage’ to account for differences in sequencing depth among samples.

### Computational tool framework and algorithm

**Fig. 1:**
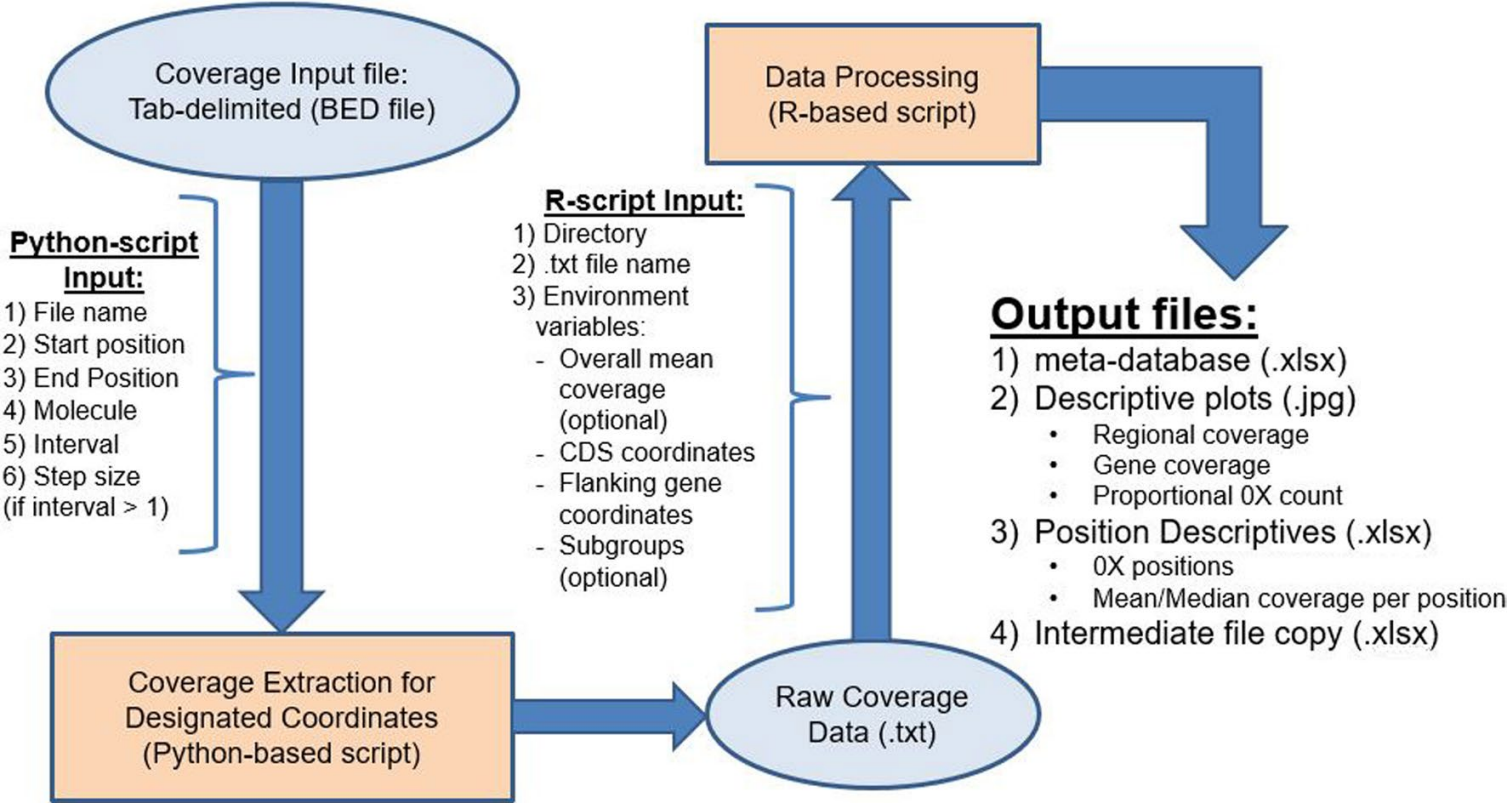
GC_3_ framework. GC_3_ extracts read coverage information and processes it into a metric database and descriptive tables/figures. Ovals denote initial/intermediate input(s). Orange rectangles denote scripts for data processing. User input parameters are needed at two junctions in the process and are listed (required and optional). (1) Python script extracts coverage data either using a “sliding window”, or coverage at every locus between user-defined start and end coordinates. Overall mean coverage between start and end coordinates can be extracted using a separate function. Output files from the python script (i.e. intermediate output) become the input into the R script, which generates metrics and relevant tables/figures. (2) User input into the R-script is required to define path (directory) to intermediate output as well as the file name, target gene coordinates, intron coordinates (if necessary), coordinates of regions of interest (e.g. flanking genes), and definition of subgroups (optional). Output from R script is Excel versions of intermediate outputs, metrics database, position descriptive database, and relevant figures

For GC_3_ to function properly, Python v3.0 and R v4.1.1 (or later versions) with the following libraries must be installed: readxl, writexl, dplyr, reshape2, and ggplot2. GC_3_ uses a Python-based script to extract read coverage information for genomic coordinates set by the user and processes these output files using an R script (Fig. 1). Following the framework, the user is required to provide input parameters at two junctions.

### Extracting target coverage data – Python-based script

Within the Python script, the user is required to provide parameter inputs depending on the desired output. To use the “sliding window” option, the user must provide: (1) name of input file, (2) start coordinate, (3) end coordinate, (4) molecule identifier containing target locus (e.g. Pf3D7_08_v3), (5) interval size (i.e. window length, in base pairs), and (6) step size (i.e. shift between windows, in base pairs). In the initial window, defined by start coordinate and interval size, the average coverage is obtained by adding read coverage across all positions and dividing by interval size. The start position is then updated by adding step size to the previous start coordinate and the process is repeated until the end coordinate is reached. The output file will report an interval’s start and end coordinates separated by a colon and flanked by apostrophes and the interval’s average read coverage separated from the coordinates by a colon (e.g. '1,290,240:1,290,740': 294.228).

If individual coverage of all positions in the interval of interest is desired, then the interval size should be set to 1, and GC_3_ will extract values for each coordinate between start and end coordinates, inclusively (step size is automatically set to 1). Output file will report the position and respective coverage (e.g. '1,372,236': 387). The intermediate output is a text file with position(s) and corresponding read coverage values.

Additionally, the user can calculate mean coverage between start and end coordinates using a separate GC_3_ function. User parameters needed are (1) name of input file, (2) start coordinate, (3) end coordinate, and (4) molecule identifier. This function is needed if the user desires to know, for example, the mean coverage over a wider region or to standardize coverage between different sets of samples (i.e., sample subgroups).

### Coverage data processing—R-based script

The user will need to input intermediate output files into the separate GC_3_’s R script to clean, and generate sample metrics and descriptive plots. At the start of the R script, the user will define (1) path to the intermediate files, (2) name of the intermediate file(s), (3) target locus’ coordinates in reference genome, (4) gene’s intron coordinates (if necessary), in reference genome, (5) position coordinates of interest (e.g. flanking gene positions), (6) list of subgroup sample identifiers and subgroup name (optional). If read coverage is to be standardized relative to coverage in a reference chromosome or chromosomal segment, then a file of mean read coverage per chromosome or segment (obtained as described above) per sample should also be defined. The GC_3_ R script will output several files, namely, (*i*) Excel version of intermediate text files, (*ii*) summary metrics: sample identifier, overall mean coverage—if mean coverage file included, mean target gene coverage, proportion of gene positions with coverage, proportion of smaller regions of interest (including coding regions, exons), deletion classification, and count of positions with 0X coverage, (*iii*) read coverage information for target gene (number of positions with zero coverage, and mean and median coverage per position over all samples), and (*iv*) descriptive plots: sliding window coverage over region of interest (i.e. subtelomeric region), all coordinates coverage over target gene positions (i.e. *hrp2* and *hrp3*), and proportion of positions with zero coverage.

Python and R scripts can be found at the Silva group’s GitHub (https://github.com/igs-jcsilva-lab) as well as a README file with detailed instructions and input examples.

### Detection of deletions in *hrp2*, *hrp3* and flanking regions

**Fig. 2:**
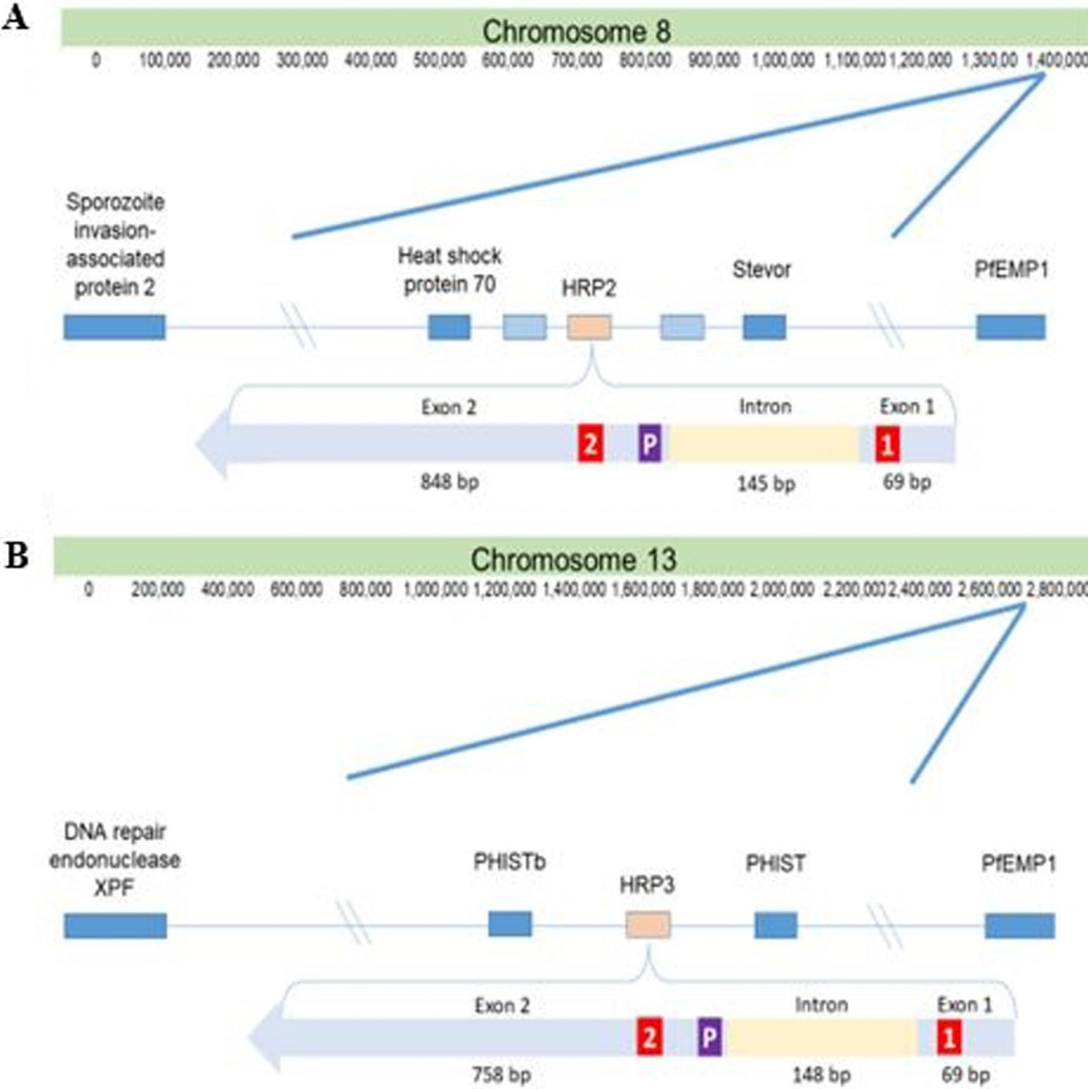
Schematic of target genomic regions, encoding *hrp2/3*.** A** Schematic of the *hrp2*-containing chromosomal region plus flanking genes (unlabeled light-blue boxes represent pseudogenes), labelled with their respective gene products, and coding regions (exon1 and exon2), their base pair length and direction of transcription. Red highlighted areas denote locations of *hrp2/3*-specific qPCR forward primer (1) and reverse primer (2), and the purple highlighted area denotes the location of the qPCR probe (P). Chromosome 08 PfEMP1 reference – Pf3D7_0833500 (multi-copy gene). ** B** Same as** A**, but for hrp3. Chromosome 13 PfEMP1 reference – Pf3D7_1373500 (multi-copy gene)

The gene structure of *hrp2* and *hrp3* in the reference 3D7 strain was obtained from PlasmoDB (www.plasmodb.org). Both *hrp2* and *hrp3* consist of two coding exons. Exon1 is 69 bp in length for both genes, and exon2 is 848 bp long in *hrp2* and 758 bp in *hrp3* (Fig. 2). The analysis of WGS data focused on subtelomeric regions of chromosome 8 (*P. falciparum* 3D7 reference strain coordinates 1,290,240–1,443,449, for a total of 153,209 bp), containing the *hrp2* coding DNA sequence (CDS) and intervening intron, and of chromosome 13 (*P. falciparum* 3D7 reference strain coordinates 2,731,041–2,892,340, for 161,299 bp), containing the *hrp3* CDS and intron. Subtelomeric coordinates were chosen to include the closest “essential” gene [25] downstream of *hrp2* or *hrp3* and farthest upstream functional gene (i.e. *Pf*EMP1-encoding *var* gene).

Metrics were generated to classify samples by presence or absence of full or partial deletions in each locus of interest. If ≤ 25% of the CDS was missing (i.e. at most 25% of the reference CDS had zero coverage) the locus was considered present with a “small deletion of uncertain functional impact” (SDUFI). If > 25% (but not 100%) of the reference CDS positions had zero coverage the sample was classified as having a partial deletion (25% < %-positions-with-zero-coverage < 100%); it was classified as having a complete deletion if all CDS positions have zero coverage. This classification is partly informed by Sepúlveda and colleagues [19], who implemented an algorithm to perform deletion calling without having to analyse the coverage profile of the entire genome. They classified deletions based on a 75% threshold of positions with ≤ 2X coverage, but may be too stringent and decreased GC_3_ thresholds as explained above to account for “SDUFIs” or partial deletions less than the 75% threshold that might impact protein detection. Deletions of flanking genes were assigned to samples if > 25% of the flanking gene’s positions reported zero coverage. Intergenic regions were excluded to reduce the effect of variable read coverages in non-coding regions.

### GC_3_ agreement with qPCR assay

A previously described *hrp2/3*-specific qPCR assay capable of detecting locus deletions in mono- and poly-clonal infections [26] was utilized to compare with the *hrp2/3* deletion genotype inferred by GC_3_. In summary, primer sequences were adapted from conventional PCR [27] to bind to conserved regions of *hrp2*, *hrp3* and an apicomplexan-specific single copy gene used as positive control, *rnr2e2* (ribonucleotide reductase R2_e2, [28]). The computational approach used by GC_3_ for the detection of *hrp2/3* deletions was compared to this *hrp2/3*-specific qPCR assay [26], using the following samples:

- NF54—Positive control
- 7G8—Positive control
- Dd2—*hrp2* absent control
- HB3—*hrp3* absent control
- 17 global samples (see Additional file 1: Table S1 for details)

The presence and classification of *hrp2/3* deletions is reported for the four laboratory reference strains mentioned above and for 17 global samples from Brazil (n = 3), Cambodia (n = 6), Mali (n = 3), Malawi (n = 4) and Thailand (n = 1). Global samples with accessible DNA material were randomly selected to represent the following GC_3_-inferred genotype subgroups: samples with no deletions, *hrp2* deletion (complete), *hrp3* deletion (complete), double *hrp2/3* deletion, low overall sample mean read coverage (< 20X), possible discordant pairs (partial deletion, with non-zero coverage in qPCR primer binding sites), and PCR primer site deletions (samples with zero coverage in qPCR primer binding site – either in *hrp2* or *hrp3*). Accession ID and subgroup stratification of global samples can be found on Additional file 1: Table S1.

### Statistical analyses

When measuring correlation between mean coverage in *hrp2/3* positions and subtelomeric or upstream/downstream gene, Spearman’s rank correlation method was used (Additional file 3). Spearman’s method accounts for non-parametric distribution and, therefore, mean coverages were not standardized [29]. R v4.1.1 program was used to conduct statistical analysis.

## Results

Sample read coverages by sliding windows of 1000 bp intervals and 500 bp step size were generated over the subtelomeric regions of chromosome 8 and chromosome 13 (sum of coverage across all positions in interval/interval length). Additionally, coverage at every position (interval = 1) was generated at every position between coordinates 1,372,236 to 1,377,299 on chromosome 8 and 2,835,756 to 2,847,557 on chromosome 13. These positions corresponded to *hrp2* and *hrp3* coordinates plus 2000 bp on either end of their respective coding regions.

### Demonstrating GC_3_ features using laboratory strains of known genotype

WGS data from reference laboratory strains were analysed to estimate *hrp2/3* coverage per bp and the proportion of positions with coverage by at least one read (≥ 1X coverage) at *hrp2* and *hrp3* coordinates, and ultimately evaluate the validity of results from GC_3_. Expected coverage was estimated using each respective subtelomeric region as reference. Overall, for each lab strain reference, excellent concordance was found between coverage values in each locus and the respective subtelomeric chromosomal regions (Table 1). Mean subtelomeric coverage of the Dd2 strain (with *hrp2* deletion genotype) was high (chromosome 8: 29X; chromosome 13: 48X), and, as expected, mean coverage at the *hrp2* positions was 0X, while *hrp3* mean coverage was 45X (with 100% of CDS coordinates with coverage > 0). The HB3 strain (*hrp3* deletion genotype) was sequenced to ~ 145X coverage (chromosome 08 and chromosome 13 subtelomeric regions with 159X and 131X coverage, respectively). Mean coverage at the *hrp3* positions was 0X (50% proportional coverage) and while *hrp2* was similar to genome-wide coverage (142X, with 100% proportional coverage of gene positions). Residual coverage may have occurred at the *hrp3* gene of HB3 despite its known deletion due to mapping of some reads originating from *hrp2* and mapping to similar but non-orthologous locations. GC_3_ correctly identified HB3 as having a *hrp3* gene deletion (Table 1). These results are similar to previously described coverage profiles of Dd2 and HB3 [19].

**Table 1:** Subtelomeric coverage, gene coverage, and coding region proportional coverage among known reference strains

Reference Strain	Mean subtelomeric read coverage (sum of coverage/bp)		Mean gene coverage (sum of coverage/bp)		Proportion of coding positions with ≥ 1X coverage	
	Chromosome 08	Chromosome 13	*hrp2*	*hrp3*	*hrp2*	*hrp3*
NF54	156.5	173.7	125.2	134.8	100%	100%
7G8	201.9	236.3	247.0	269.9	100%	100%
NF135.C10	35.3	42.7	36.9	41.7	100%	97%
NF166	280.0	328.4	295.7	344.6	100%	100%
Dd2	29.3	48.3	0.0	45.0	0%	100%
HB3	159.3	130.5	142.2	0.35	100%	50%

### Plotting of read coverage in subtelomeric region of select reference strains

**Fig. 3:**
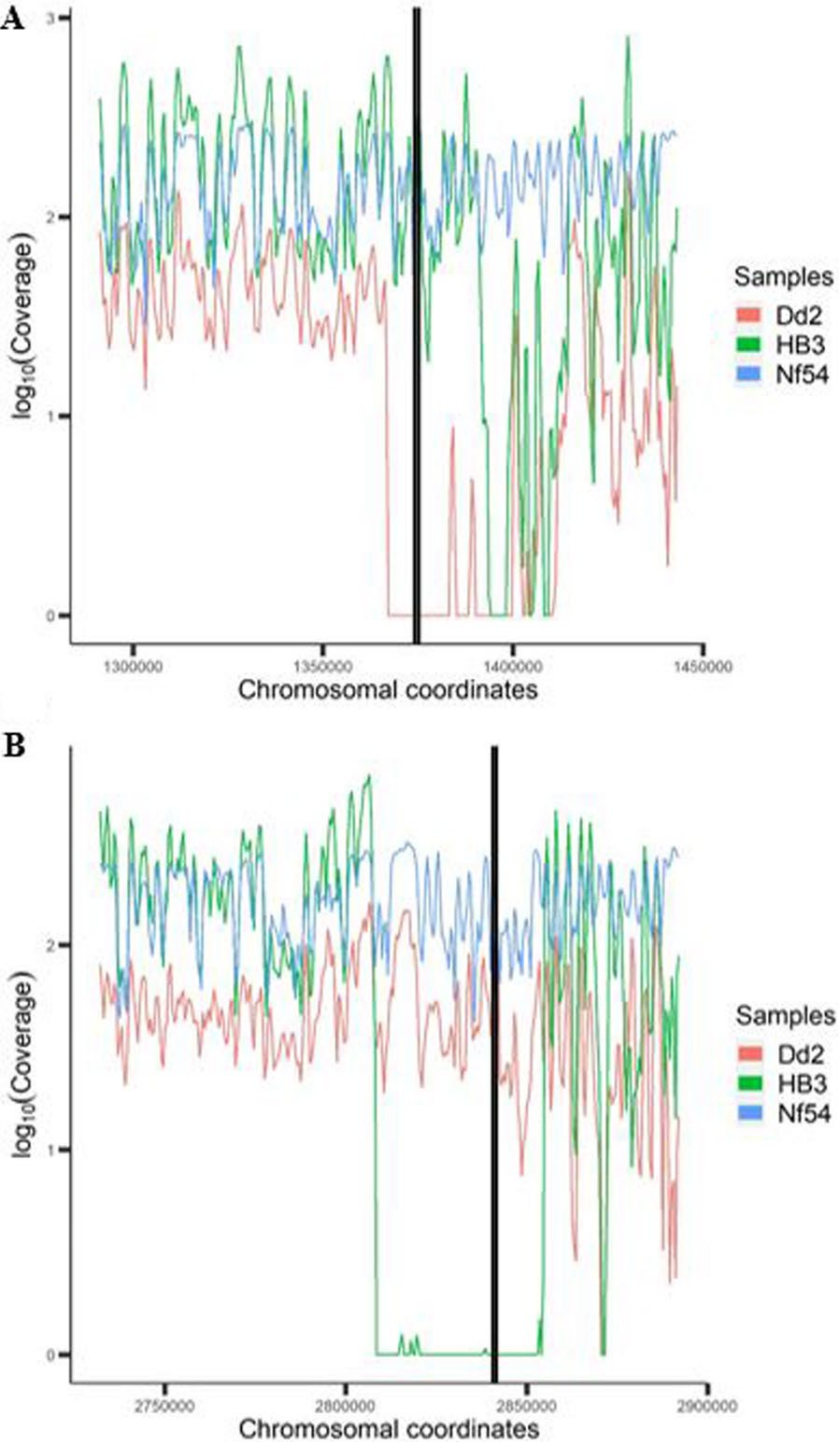
Subtelomeric read coverage distribution plots. Log_10_ of subtelomeric read coverage for reference strains NF54 (*hrp2* and *hrp3* present), Dd2 (*hrp2* absent) and HB3 (*hrp3* absent). Coverage was measured using a 1000 bp sliding window (i.e. interval) with a 500 bp “step” between windows. A. Chromosome 08 subtelomeric region coverage (black lines denotes *hrp2* coding positions 1,374,236–1,375,299). B. Chromosome 13 subtelomeric region coverage (black lines denotes *hrp3* coding positions 2,840,727–2,841,703)

GC_3_ can create plots of the sliding window findings in order to provide a visual perspective of the target region. To illustrate the coverage data provided in Table 1, the subtelomeric regions containing *hrp2* and *hrp3* of reference strains Dd2, HB3 and NF54 were plotted (Fig. 3). Results were normalized on a log scale to better visualize large fluctuations in coverage generated by whole genome shotgun sequencing. Sliding window plots confirm validation results of Dd2, HB3 and NF54, and clearly illustrate coverage for each respective strain. On chromosome 08, coverage of the Dd2 strain decreases to zero for several thousand base pairs that include the *hrp2* locus, whereas NF54 and HB3 have high coverage in the same region. Noticeably, in the HB3 strain, a section of the subtelomeric region upstream of the *hrp2* CDS has poor coverage (near position 1,400,000 on chromosome 8). This section of poor coverage would not impact *hrp2* presence/absence, and could be due to the presence of one or more deletion(s), or to poor mapping. Poor read mapping can occur in the subtelomeric regions for several reasons, including the presence of multiple members of highly variable multigene families (*var*, *stevor* and *rifin*) that differ between strains or to the presence of low complexity regions. On chromosome 13, it is the HB3 strain that has several thousand base pairs with little or no coverage, including the *hrp3* locus, whereas NF54 and Dd2 coverage remains high. GC_3_ visuals showed no deletions in NF54, a complete *hrp2* deletion in Dd2 and a large section of little to no coverage at the *hrp3* locus in HB3, respectively.

### GC_3_ agreement with a hrp2/3-specific qPCR assay on field samples

**Fig. 4:**
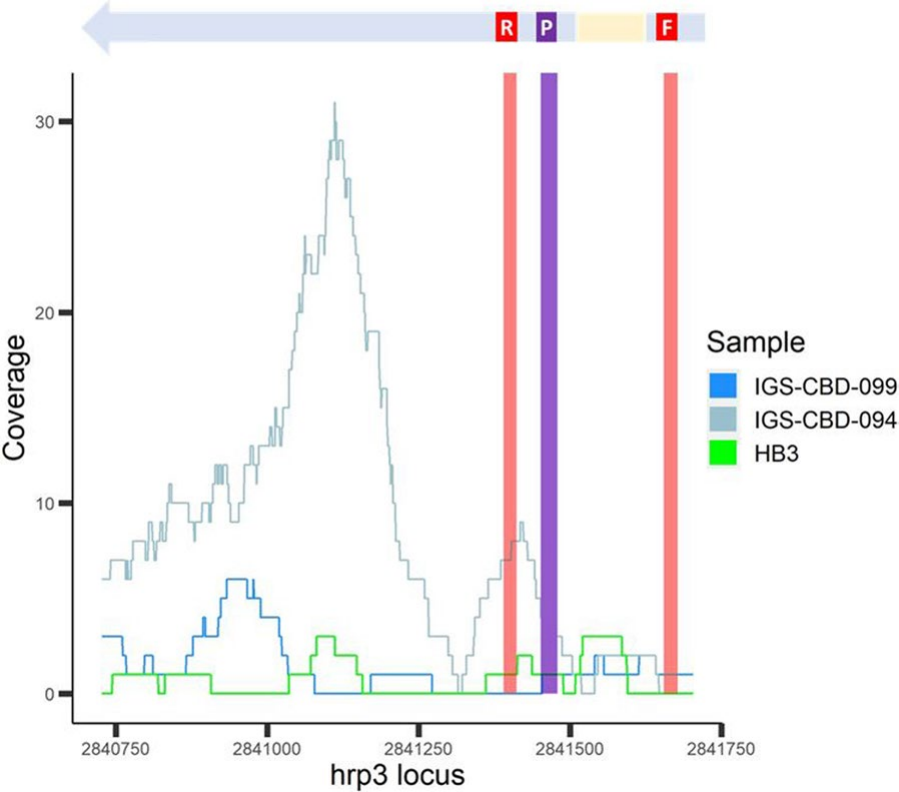
Coverage of *hrp3* among select validated samples. Coverage plot of *hrp3* locus of validated samples IGS-CBD-099, IGS-CBD-094 and strain HB3 (known *hrp3* deletion genotype) with *hrp3* schematic representation above the plot. Red areas denote the primer binding sites, and purple area denotes the probe binding site of the *hrp2/3*-specific qPCR assay. GC3 assigned IGS-CBD-099 as *hrp3*-absent; however the 5′ end of the gene was amplified by qPCR. As a comparator, IGS-CBD-094 is a similar sample (apparent deletion at qPCR primer binding site in exon 1) that GC_3_ assigned as *hrp3*-present. HB3 is a laboratory reference strain known to be missing the *hrp3* locus. It is important to note some position with non-zero coverage in HB3, which suggests that non-orthologous reads from HB3 map the *hrp3* locus of Pf3D7

A subset of global samples (n = 17) and reference strains (n = 4) underwent qPCR specific for *hrp2* and *hrp3* to compare with GC_3_ results. Two among the selected global samples were excluded due to low parasitaemia resulting in very low or no detection of the positive control gene by qPCR (Cq threshold cutoff = 37.5). There was very good agreement between GC_3_ (computational) and qPCR assay results. Out of four references strains and remaining 15 global samples, GC_3_ matched qPCR results 19/19 (100%) for *hrp2* and 18/19 (94.7%) for *hrp3* (Table 2). Only one sample (IGS-CBD-099) had a discordant result between methods. In particular, for this sample, GC_3_ classified it as having a partial deletion at the *hrp3* locus, and coverage assessment with base-pair granularity suggested partial lack of read coverage, including the exon 2 primer binding region, between 2,841,390—2,841,412 (Fig. 4). It is noteworthy that the average coverage in this region is very low (~ 1X), however coverage is high for the corresponding chromosomal subtelomeric and core regions (~ 124× and ~ 143×, respectively). On the other hand, the qPCR assay was positive for *hrp3* (Cq = 25.4). Taken together, the results suggest the sample has a partial deletion at the *hrp3* locus, which does not encompass the qPCR primer binding regions, but that is possibly close enough to the binding site of the primer in exon 2 to interfere with read mapping in that region.

**Table 2:** Agreement between GC_3_ deletion assessment results on global samples and *hrp2/3*-specific qPCR assay^a^

Sample name	Country	Phenotype subgroup	*hrp2* ^b^	*hrp3* ^b^	*rnr2e2*
					(control gene)
			GC_3_/PCR	GC_3_/PCR	PCR
7G8	Reference (Brazil)	Control	Present/Present	Present/Present	Present
NF54	Reference	Control	Present/Present	Present/Present	Present
Dd2	Reference (Laos)	Control—*hrp2* deletion	Absent/Absent	Present/Present	Present
HB3	Reference (Honduras)	Control—*hrp3* deletion	Present/Present	Absent/Absent	Present
IGS-BRA-017sA	Brazil	No deletions	Present/Present	Present/Present	Present
IGS-THL-017	Thailand	No deletions	Present/Present	Present/Present	Present
IGS-BRA-021	Brazil	No deletions	Present/Present	Present/Present	Present
IGS-CBD-026	Cambodia	No deletions	Present/Present	Present/Present	Present
IGS-CBD-031	Cambodia	*hrp2* deletion (complete)	Absent/Absent	Present/Present	Present
IGS-MLI-036	Mali	*hrp3* deletion (complete)	Present/Present	Absent/Absent	Present
IGS-BRA-001sA	Brazil	Double *hrp2/3* deletion	Absent/Absent	Absent/Absent	Present
IGS-CBD-008	Cambodia	Low coverage sample	Present/Present	Present/Present	Present
IGS-MWI-254sA	Malawi	Low coverage sample	Present/Present	Present/Present	Present
IGS-MWI-251sA	Malawi	Low coverage sample	Present/Present	Present/Present	Present
IGS-MLI-039	Mali	*hrp2* discordant pair	Present/Present	Present/Present	Present
IGS-MLI-031	Mali	*hrp3* discordant pair	Present/Present	Present/Present	Present
IGS-CBD-034	Cambodia	*hrp2* PCR primer deletion	Present/Present	Present/Present	Present
IGS-CBD-094	Cambodia	*hrp3* PCR primer deletion	Present/Present	Present/Present	Present
IGS-CBD-099	Cambodia	*hrp3* PCR primer deletion	Present/Present	Absent/Present	Present

^a^ Cells in purple and blue denote agreement between GC_3_ and qPCR results (i.e. "PCR"); red denotes disagreement between methods

### Comparison of GC_3_ output using Kenyan and Peruvian sample sets previously genotyped for hrp2/3 deletions

Previously, a subset of Kenyan samples (n = 27) was genotyped for *hrp2/3* deletions, with two and one deletions identified in *hrp2* and *hrp3*, respectively [19] (Table 3). In addition, Sepúlveda and colleagues also identified no *hrp2* deletions among twelve Peruvian samples and two samples with deletions in *hrp3* [19]. In that study, the criterion used to call deletions was > 75% of gene positions with ≤ 2X coverage [19]. To determine how GC_3_ performed on a similar set of samples SRA samples were downloaded from the same time point from Kenya (n = 57, including 24 from [19]) and Peru (n = 11, including 7 from [19]). For *hrp3*, the same number of deletions were identified as reported previously [19]. However, the *hrp2* were discordant. One complete deletion was identified among Kenyan samples, and one partial *hrp2* deletion among the Peruvian samples, which differs from previous reporting. Among the Kenyan samples, a previous study reported two *hrp2* deletions [19], one of which was also identified by GC_3_. Whereas the discordant Peruvian sample was only identified to have a deletion by GC_3_. The difference in assessment is likely due to differences in the criteria used between GC_3_ and that used by Sepúlveda and colleagues to call deletions.

**Table 3:** Deletion identification and classification of previously genotyped samples

Country	n	*hrp2*					
		No Deletion	GC_3_ Deletion Classification (Partial/Complete)^a^	ex1-/ex2 + ^b^	ex1 + /ex2-^b^	ex1-/ex2-^b^	Previous Genotype (Deletion/Total)^c^
Kenya	59	58	0/1	0	0	1	2/27
Peru	11	10	1/0	1	0	0	0/12

Country	n	*hrp3*					
		No Deletion	GC_3_Deletion Classification (Partial/Complete)^a^	ex1-/ex2 + ^b^	ex1 + /ex2-^b^	ex1-/ex2-^b^	Previous Genotype (Deletion/Total)^c^
Kenya	59	58	1/0	0	0	1	1/27
Peru	11	9	2/0	0	1	1	2/12

^a^*hrp2/3* deletions assigned to isolates with > 25% CDS positions with zero coverage

^b^Exon absence (-) assigned to isolates if > 25% exon positions have zero coverage. ( +) signifies exon is present

^c^Previous *hrp2*/3 deletion genotype results from Sepúlveda and colleagues. Deletions called for samples with > 75% of coding region with ≤ 2X coverage [19]

### Analysis of novel samples for hrp2 and hrp3 deletions

All global samples used in the study (n = 1114) were examined for *hrp2/3* deletions (Additional file 1: Table S2). Cambodian (n = 127) and Brazilian (n = 20) samples were further visualized in more detail at the subtelomeric regions of interest (Additional file 1: Figure S2) and examined for *hrp2/3* exon presence/absence since they have not previously been described (Table 4). Although *hrp2/3* have not been described for these samples, computational results are comparable to previous estimates in each respective region, where deletions have been previously observed [19, 30]. Among Cambodian samples collected in 2009–2011, there were one *hrp2* deletion, eight *hrp3* deletions, and two *hrp2/3* double deletions (both *hrp2* and *hrp3*), with frequencies in this sample set of 0.8%, 6.3% and 1.6%, respectively. All *hrp2* deletions corresponded to absent exons (> 25% zero coverage positions on both exons), but were classified as two partial and one complete *hrp2* deletion as two samples still had coverage in a low proportion on *hrp2* positions. Deletions of *hrp3* among Cambodian samples were classified as seven partial and three complete deletions. Among Brazilian samples collected in 2016, four had *hrp2/*3 double deletions, and eleven with *hrp3* deletions, corresponding to frequencies of 20% and 55% respectively. Of the four *hrp2* deletions, two were partial deletions, and two were complete *hrp2* deletions. Of the 15 *hrp3* deletions, one was a partial deletion on exon 2, and 14 were complete *hrp3* deletions.

**Table 4:** Deletion identification and classification of undescribed samples

Country	n	*hrp2*				
		No Deletion	Deletion Classification (Partial/Complete)^a^	ex1-/ex2 + ^b^	ex1 + /ex2-^b^	ex1-/ex2-^b^
Cambodia	127	124	2/1	0	0	3
Brazil	20	16	2/2	0	1	3

Country	n	*hrp3*				
		No Deletion	Deletion Classification (Partial/Complete)^a^	ex1-/ex2 + ^b^	ex1 + /ex2-^b^	ex1-/ex2-^b^
Cambodia	127	117	7/3	0	2	8
Brazil	20	5	1/14	0	1	14

^a^*hrp2/3* deletions assigned to isolates with > 25% CDS positions with zero coverage

^b^Exon absence (-) assigned to isolates if > 25% exon positions have zero coverage. ( +) signifies exon is present

### Quantifying flanking region deletions among Cambodian samples

Deletions at the *hrp2/3* positions may extend to flanking genes, possibly with additional impact on overall parasite fitness. Therefore, an option in the GC_3_ R-script was built in to assign deletions of flanking genes. To determine whether deletions extend into these flanking coding regions, a table was generated for Cambodian samples, where samples with > 25% gene positions with zero coverage in upstream and downstream flanking regions were classified as having a locus deletion (Table 5). Of note are the observations that the presence of a *hrp2/3* deletion is not always associated with deletions in flanking genes and, conversely, deletions in flanking genes are not always associated with *hrp2/3* deletions. A subset of Cambodian samples has been plotted to illustrate flanking gene coverage as it relates to *hrp2* or *hrp3* (Additional file 1: Figure S3). Results suggest that deletions can occur independently in *hrp2* (or *hrp3*) and their respective flanking genes.

**Table 5:** Frequency of deletions in *hrp2/3* flanking genes, among Cambodian samples

Flanking gene^b^ (Upstream/Downstream)^c^	*hrp2*^a^		*hrp3*^a^	
	Present	Absent	Present	Absent
Present/Present	124	2	113	3
Absent/Present	0	1	4	0
Present/Absent	0	0	0	1
Absent/Absent	0	0	0	6
Total	124	3	117	10

^a^Deletion assigned to samples if > 25% of coding region positions had zero coverage

^b^Deletion of flanking genes assigned to samples with > 25% gene positions with zero coverage

^c^For *hrp2*, an upstream gene was a STEVOR family gene, and a downstream gene encoded heat shock protein 70. For *hrp3*, upstream and downstream genes were PHIST-encoding genes of unknown function

### Temporal comparison of standardized coverage of hrp2/3 positions

**Fig. 5:**
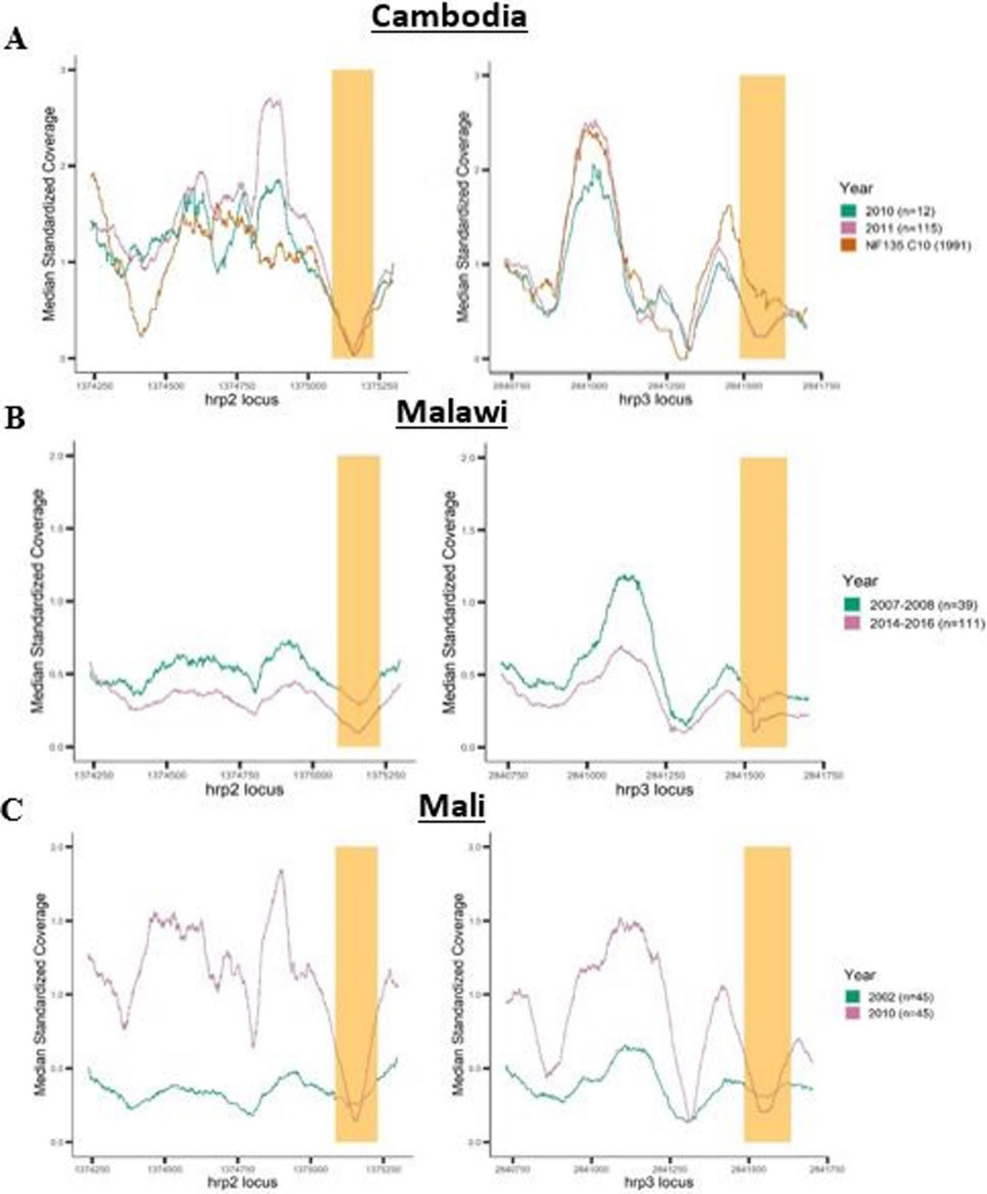
Median of standardized coverage between longitudinal subgroups. Median of standardized coverage [SUM (Coverage/Subtelomeric Mean Coverage)/ Total Samples] in *hrp2* and *hrp3* grouped by year of sample collection (Year). Countries include A. Cambodian *hrp2* and *hrp3* positions (n = 127), B. Malawi *hrp2* and *hrp3* positions (n = 150), C. Mali *hrp2* and *hrp3* positions (n = 90). Tan shading marks intron positions of target gene, whereas unshaded areas are exon positions (*hrp2*—> exon 1: 1,375,299–1,385,231; intron: 1,375,230–1,375,085; exon 2: 1,375,084–1,374,236; *hrp3*—> exon 1: 2,841,703–2,841,635; intron: 2,841,634–2,841,486; exon 2: 2,841,485–2,840,727). Analysis and figures were generated using R v4.1.1

Coverage plots of *hrp2/3* coordinates were generated for Cambodian, Malawian, and Malian samples to demonstrate: (1) magnified plots of only *hrp2/3* positions and (2) differences in relative depth of coverage between samples collected at different time points (Fig. 5). Cambodian samples were collected in Battambang, Pailin, Koh Kong, Kampot, Kampong Speu, Oddar Meanchey, Preah Sihanouk, and Preah Vihear districts from volunteers aged 18–65 years in 2010 and 2011, and then sequenced at IGS with 100 bp paired-end Illumina reads [31]. NF135.C10 was cultured in the laboratory and sequenced at IGS with 150 bp paired-end reads. Two datasets of Malawian samples were collected in 2007–08 and 2014–16. Samples from 2007–08 were collected during a malaria drug study in Ndirande, outside Blantyre, from children 6 months to 5 years of age and sequenced at IGS with 150 bp paired-end reads [32]. Samples collected in 2014–16 are from a cohort study of malaria incidence in Chikwawa, south of Blantyre, where samples from volunteers aged 2–8 years were sequenced at IGS with 150 bp paired-end reads [22]. Comparisons were also made between two Malian datasets from 2002 and 2010, both collected in Bandiagara, Mali. Samples from 2002 are from a case/control study of severe malaria among 3 months to 14 year old volunteers and were sequenced at IGS with 100 bp paired-end reads [33]. Malian samples collected in 2010 are from a cohort study of malaria incidence among volunteers aged 1–5 years and sequenced at IGS using 150 bp paired-end reads [34]. It should be noted that comparing these datasets are for illustrative purposes only, since the extent to which relatively small samples sizes and potential confounders (including sampling location and strategy, sample independence, sequencing approach, read length) impact observed deletion frequency is unknown. To account for differences in sequencing depths between samples, coverage values were standardized. For each sample, site or locus coverage were divided by the expected coverage, obtained from mean coverage in subtelomeric region in which each locus is located (see Methods for coordinates). Standardized coverage of ~ 1 shows locus coverage similar to subtelomeric mean coverage. A strong, positive correlation between subtelomeric and *hrp2/3* gene coverage justifies the use of this standardization approach (Additional file 1: Figures S4 and S5). A decrease in standardized coverage over time would suggest an increase in frequency in *hrp2/3* deletions. To avoid undue impact of outlier standardized values, median standardized coverage was plotted per group.

Standardized read coverage for Cambodian samples was plotted alongside standardized coverage for the geographically representative strain NF135.C10 (Fig. 5A), and showed that the uneven standardized coverage of NF135.C10 is mirrored in the clinical samples. This suggests there are sequence-inherent properties that impact sequencing or mapping success. (see Additional file 2: Table S3 for descriptive coverage of each sample). In contrast, Malawian samples collected in 2014–2016 had lower median standardized coverage than the sample set collected in 2007–2008 (Fig. 5B), everything else being equal, this would suggest an increase in *hrp2* and *hrp3* deletions between the two time points. Interestingly, median standardized coverage is low (< 1) in both time points, showing that read coverage in the target genes is half of that in the respective subtelomeric regions. The majority of Malawian samples underwent sWGA (n = 139) prior to sequencing (Additional file 2: Table S4) which may explain the lower standardized coverage as compared to standardized coverage of directly sequenced samples (Additional file 1: Figure S6). Finally, Mali samples from 2002 showed lower standardized coverage than 2010 samples on both *hrp2* and *hrp3* positions (Fig. 5C). All Malian WGS data was obtained by direct sequencing of total DNA from venous blood, using a similar protocol [22, 33] with high sequence coverage in the core genomes (Additional file 2: Table S5), a strong suggestion that the quality of WGS data did not contribute to the observed difference. However, it remains unclear if the datasets are directly comparable as differences in, for example, sample strategy/bias and read length could distort observed frequencies [22, 33, 34]. Mali results highlight the potential impact of read length in some of the observed results. Lower coverage may be due to mapping ambiguity in smaller read datasets. Overall, figures offer a visual perspective between different time points as monitoring of *hrp2/3*-deletions become crucial in the possibility of their expansion; however, caution should be applied since the factors that influence WGS coverage need to be considered as part of any interpretation.

### Count of positions with zero coverage

**Fig. 6:**
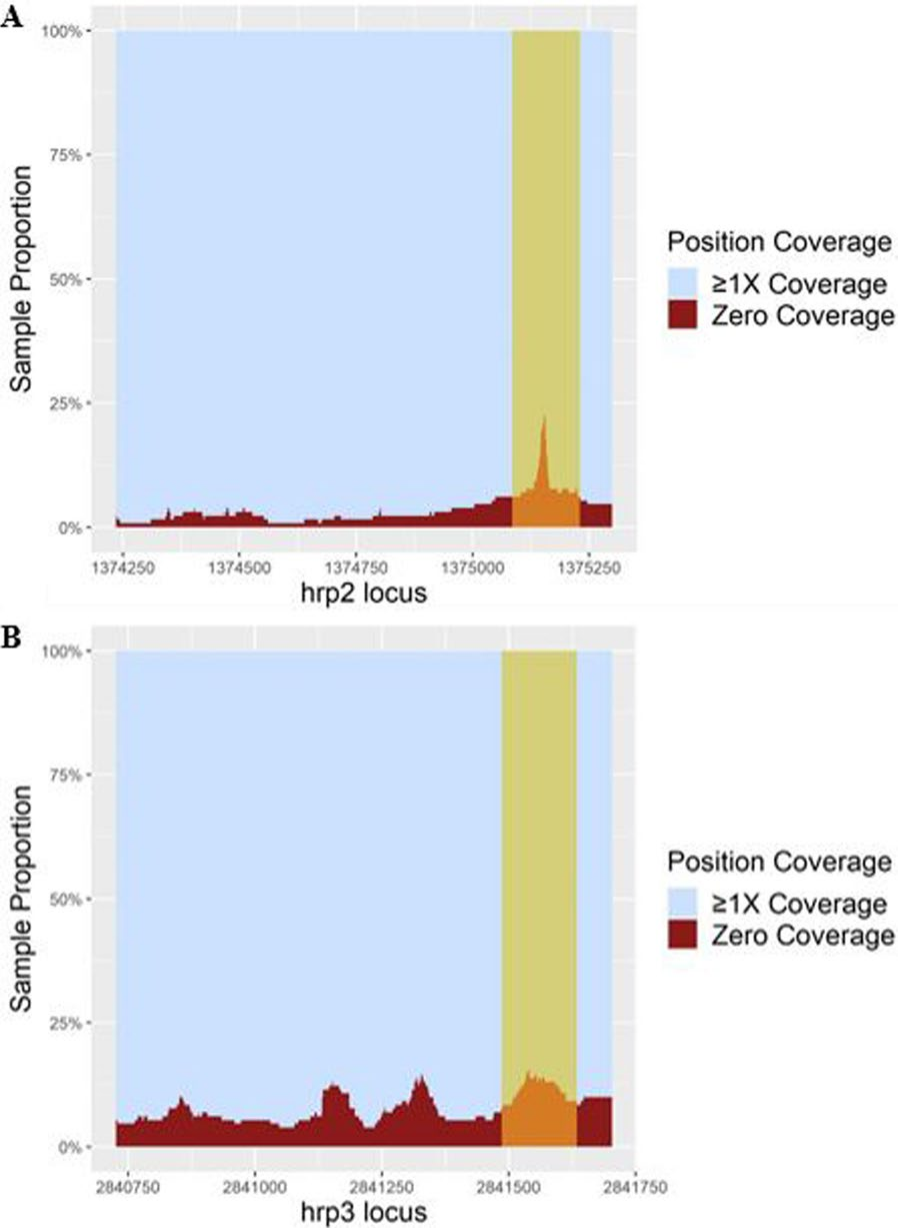
Proportional count of zero coverage positions at the *hrp2/3* positions Proportional sample count of *hrp2* and *hrp3* gene positions with zero coverage vs. positions with ≥ 1X coverage among Cambodian samples (n = 127). A. shows count of zero coverage positions among *hrp2* positions, and B. shows count of zero coverage positions among *hrp3* positions. The tan section on each plot represents the intron region of each respective gene. Analysis and figures were generated using R v4.1.1

To provide a clearer illustration of the proportional frequency of gene coordinates with zero coverage and the location of those positions along the locus, a view of *hrp2* and *hrp3* gene positions by proportional counts of no coverage (0X coverage) *vs.* coverage (≥ 1X coverage) was generated for Cambodian samples (Fig. 6). Additional file 1: Figure S7 provides the same proportional counts of zero coverage per position for Malawian and Mali samples. Among Cambodian samples, there is a clear increase in 0X coverage positions at the intron regions (*hrp2* intron: 1,375,232–1,375,083; *hrp3* intron: 2,841,636–2,841,484) relative to exon coverage. This is to be expected, as the length (145–148 bp) and the nucleotide composition of these *Pf* introns (*hrp2* AT%: 91%; *hrp3* AT%: 91.2%) prevent unambiguous mapping of 101 bp-long reads centered in the middle of the intron. On *hrp3* positions, there are also two spikes in zero coverage positions on either side of coordinate 2,841,250, likely due to differences in Cambodia samples compared to *Pf*3D7 reference, such as indels or rapidly evolving sequence motifs among genetically similar Cambodian strains, which prevent read mapping in a subset of samples. That read mapping pattern is also observed in the troughs in *hrp3* coverage plot in Fig. 5A, for samples collected in 2010 and 2011 (but curiously not in Pf NF135).

## Discussion

Next-generation short-read WGS data has the capability to provide detailed genotype information, but often necessitates a good understanding and use of bioinformatics tools and packages. GC_3_ was developed to be a user-friendly computational tool to (1) extract coverage profiles of target genome regions, (2) provide interpretable results regarding location and frequency of deletions, (3) classify samples according to the type of gene deletions, and (4) validate large-scale, qPCR-based, studies conducted to inform NMCPs concerning frequency of copy number variants (including deletions) in genes of translational importance. In this study, it is demonstrated that GC_3_ can be used for these purposes by applying it to *Plasmodium falciparum* genome segments, specifically the regions containing *hrp2* and *hrp3* genes. Most NMCPs in malaria-endemic settings rely on HRP2-based RDTs for day-to-day diagnosis in both clinical and field settings. There is evidence of recent expansions of *P. falciparum* strains lacking HRP2, a cause for concern as stated by the WHO [1, 20, 35, 36]. Given the continuing decrease in sequencing costs and the widespread generation of WGS data, computational tools, such as GC_3_, that take advantage of such data to efficiently assess the presence of *hrp2*- or *hrp3*-deletion strains, provides a valuable monitoring resource to researchers and public health professionals concerned with malaria RDT effectiveness.

This work demonstrated the validity and utility of the GC_3_ tool to assess *hrp2* and *hrp3* deletion frequencies in *P. falciparum* sample sets, as well as to carefully characterize those deletions in individual samples. The sliding window capability of GC_3_ provided a wider view of large deletions in reference lab strains (i.e. Dd2 and HB3) while adjusting for fluctuations in subtelomeric read coverage and allowed for visually-friendly figures. The option of extracting and plotting every chromosome position within an interval allowed for a magnified view of target loci, and better illustrated details in coverage within the target gene. GC_3_’s genotype results were validated against previously reported genotypes of *P. falciparum* laboratory reference and representative strains, and similar, publicly available sample sets from Kenya and Peru, where *hrp2/3* deletions have been observed [12, 19, 37]. Analysis of samples from Cambodia and Brazil demonstrate GC_3_’s capability to process novel WGS data from regions other than West Africa, the location of origin of PfNF54, the parental isolate from which the reference 3D7 was cloned [38, 39]. Results are consistent with previous estimates of *hrp2/*3 deletions among each respective country [20, 30, 40]. Of note is the high prevalence of all deletions, and especially *hrp3* deletions among Brazilian samples. Deletions in *hrp2/3*, and reports of high deletion prevalence, were first observed in the American continent [8, 9, 17, 19, 40–42], especially in the *hrp3* locus [8, 43]. Overall, GC_3_ can appropriately process and analyse publicly available WGS datasets from a variety of genomic studies.

Additional comparison against a *hrp2/3*-specific qPCR assay demonstrated very good reliability of GC_3_’s capability. Although there was one discordant result between tools, this may be a reflection of GC_3_’s sensitivity and the qPCR assay’s difficulty to detect partial deletions, as these are only detected by the qPCR-based assay if they overlap the primer-binding or amplicon sites. A potential challenge for GC_3_ are the samples with a very low amount of parasite DNA resulting in genomic libraries of substandard quality and overall low depth of coverage and uneven representation of the loci of interest in the genomic library and/or among the WGS data, leading to significant regions of the *hrp2/3* loci with zero coverage (and then a ‘deletion’ assessment by GC_3_), despite the loci being present in the genome. However, this situation was not observed in this study. In the specific case of discordant results in a sample from Cambodia, the sample had very high coverage at the core and subtelomeric regions. In general, partial deletions present a challenge since some cases have shown a qPCR assay can amplify part of *hrp2/3*, but corresponds to false negative RDT diagnoses [44, 45]. Ultimately, there were very few such samples, so their impact on overall results is considered minimal. Although beyond the scope of this study, further examination of *hrp2/3* partial deletions, their specific location within the locus and their respective RDT diagnosis may provide valuable information regarding the most appropriate criteria and thresholds to accurately identify gene deletions with a functional phenotype, i.e., those deletions that abrogate protein expression.

The application of GC_3_ to describe and visualize patterns of partial deletions makes it a valuable resource for research purposes. By providing exact genomic coordinates that lack read coverage, GC_3_ may inform on genomic sequence backgrounds more prone to such mutations and eventually deletions that prevent RDT detection. Defining exact locations of deletions allows the user to determine whether the observed deletions can be explained by a single deletion event followed by lineage expansion, or whether multiple events need to be invoked to explain the observations. This examination of the evolutionary history of gene deletions can provide insights into mechanisms and rate of origin of indels and allow for improved monitoring of target genes.

Among Cambodian samples, read coverage in flanking genes was further analysed and demonstrated that *hrp2/3* deletions can be restricted to just the locus proper, or extend to flanking genes, but without a discernible pattern. These results are consistent to previous reports [19, 20, 43, 46, 47]. Overall, GC_3_ reported similar results in deletion frequency and classification trends within and among global malaria-endemic regions.

Utilizing the function of GC_3_ to extract all positions (i.e. interval = 1, step size = 1) and plotting *hrp2/3* coding positions only, coverage is clearer and subsets of samples can be compared, if desired. The greater difference in median relative coverage was visualized between the two sample sets collected in Bandiagara, Mali. Samples collected in 2002 had lower standardized coverage than those collected in 2010, in both *hrp2* and *hrp3*. Were these samples comparable, this result would be unexpected, since the frequency of deletions is expected to have increased over time, due to the selection imposed by parasite detection by RDT. Interestingly, *hrp2* deletions had already been observed in Mali in the late 1990s prior to significant RDT use in the country, indicative of deletion recurrence or persistence in the population at the time despite the absence of pressure from RDT usage [48]. In fact, random polymorphisms occur naturally particularly in the subtelomeric region, including large deletions, without evolutionary pressure [49]. Further, the fitness cost associated with *hrp2* loss is not significant, although a small cost appears to be associated with *hrp3* deletions [3, 19]. However, too many potentially confounding factors exist between the two sample sets to appropriately interpret results as being indicative of a significant change in frequency of these gene deletions. Even slight differences, apparently innocuous, between sample sets, like read length (100 bp in Mali 2002 samples and 150 bp in Mali 2010 samples), could have an impact when comparing sample sets. Other factors include how malaria-positive cases were detected, study objective (case–control study vs. cohort study), and sample collection strategy (passive vs. active). A carefully controlled study using large sample sizes would be necessary to draw definitive conclusions. Ultimately, this observation showcases GC_3_’s capability to visualize coverage patterns between sample sets and how other factors can impact deletion rate at these loci.

Closer examination of the proportional coverage at each *hrp2/3* position among Cambodian samples, revealed how mapping artifacts can result in no coverage, and potentially confounding results. On *hrp2*, zero coverage positions increase and then spike around the intron region which, as mentioned before, is likely due to the intron’s high AT content [50] that can cause challenges for read mapping. Interestingly, there were two spikes in zero coverage positions on the *hrp3* locus. Examination of a previous whole genome sequence-based hierarchical cluster analysis of the same Cambodian samples [31] revealed that the majority of samples contributing to one or both peaks belong to the same Cambodian subpopulation and hence share a similar genetic background. Ultimately, the figure offers a useful preliminary view of the *hrp2/3* genes and their characteristics.

Some limitations exist when interpreting GC_3_’s results, particularly when comparing sample sets. In this case, it is critical to ensure that sample sets are directly comparable (similar sampling location, collection design and protocol, sample processing and sequencing approach, etc.) or else that interpretation of results is robust to potential confounding factors. In such studies, GC_3_ is most useful when WGS data is all that is available, and biological material has been exhausted. Pertaining to GC_3_’s results, the quality of computational results is influenced by the depth of the *P. falciparum* sequencing data, as measured by the total number of reads mapped to the reference genome. In the case of *P. falciparum*, it is considered good coverage data if the percentage of genome with coverage asymptotes at ~ 12 million 100-bp reads mapped to the parasite genome, averaging ~ 52X coverage genome-wide. It is also recommended that GC_3_ is used to calculate mean coverage over the broader region/chromosome where the target gene is located to estimate expected coverage. Further, high-quality WGS data results obtained with well-described DNA extraction methods and sequencing methods, either by direct sequencing or sWGA [22, 24], and established quality control and filtering protocols should be used when comparing samples from different studies. When comparing standardized coverage between direct and sWGA sequence data, direct sequencing achieves more uniform coverage due to the inefficient amplification on the subtelomeric region by sWGA primers [24], but sWGA still provides good coverage at *hrp2/3* gene positions (Additional file 1: Figure S6). Even with high-quality data, polyclonality adds another layer of complexity, especially in high transmission settings, where these are most common, since the presence of multiple strains can mask the lack of coverage at a target gene absent in some but not all strains [18]. This factor would need to be considered particularly if GC_3_ is the only method being used to assess for the presence of deletions. The deletion criteria can be easily adjusted by the user to be more stringent depending on their purposes, much like the deletion criteria used in the comparator study [19]. Despite the limitations, GC_3_ appropriately processed *hrp2/3* coverage data and classified deletions. Its utility can be extended to analyse and visualize coverage data of any target gene on any pathogen.

### Summary statement

Overall, validation of GC_3_ to extract and process WGS data was successful when comparing with expected results using reference strains, well-described samples and a *hrp2/*3-specific qPCR assay. Following the criteria for identifying deletions, the results agreed with previous estimations of *hrp2/3* deletion frequency in each respective country. Apparent in the results is the level of detail that can be extracted from short-read WGS data and viewed using a comprehensive computational tool. Although challenges persist in ensuring high-quality WGS data and achieving similar coverage among low parasitaemia samples using sWGA, GC_3_’s results are expected to be fairly robust. Further investigation of the partial deletions threshold that results in a false negative RDT diagnosis is needed to validate the deletion criteria. Ultimately, groups investigating a target gene’s coverage can use GC_3_ to efficiently generate translatable results and figures to understand and interpret broad patterns using hundreds to thousands of previously generated genomic datasets.

## Supplementary Information

**Additional file 1: Figure S1.** Distribution of global isolates including reference strains NF54 (West Africa), 7G8 (Brazil), NF135.C10 (Cambodia), NF166 (Guinea), DD2 (Laos) and HB3 (Honduras). Figure created using Mapchart.net.** Figure S2.** Subtelomeric read coverage distribution plots.** Figure S3.** Cambodian sample subset of hrp2/3 and flanking genes.** Figure S4.** Scatter plots of subtelomeric mean coverage vs. mean coverage of respective HRP-encoding locus.** Figure S5.** Scatter plots of mean downstream/upstream gene coverage vs. mean coverage of respective HRP-encoding locus.** Figure S6.** Median standardized coverage by direct sequencing vs sWGA.** Figure S7.** Proportion of hrp2/3 positions with 0X vs ≥1X coverage for Malawi and Mali samples.** Table S1.** List of samples that underwent hrp2/3-specific qPCR assay.** Table S2.** GC3 deletion assignments for hrp2/3 per country.

**Additional file 2: Table S3.** Summaries of Cambodian sample gene and chromosomal coverages grouped by year of collection.** Table S4.** Summaries of Malawian sample gene and chromosomal coverages grouped by year of collection. **Table S5.** Summaries of Malian sample gene and chromosomal coverages grouped by year of collection.

**Additional file 3. **Samples used for analyses in the manuscript "“Gene Coverage Count and Classification” (GC3), a coverage assessment tool, and its application to identify and classify histidine-rich protein 2 and 3 deletions in Plasmodium falciparum using short-read whole genome sequencing data".

## Data Availability

The datasets from field studies are available from public repositories, with access information reported previously [22].

## Abbreviations

NMCP: National Malaria Control Programme
RDTs: Rapid Diagnostic Tests
*hrp2/3*: Loci encoding histidine-rich proteins 2 and 3
WGS: Whole Genome Sequencing
sWGA: Selective Whole Genome Amplification
SRA: Sequence Read Archive
BAM: Binary sequence Alignment/Map
GATK: Genome Analysis Toolkit
BED: Browser Extensible Data
bp: Base pair
CDS: Coding DNA sequence
