# Supplementary material for: Gene Coverage Count and Classification (GC3): a locus sequence coverage assessment tool using short-read whole genome sequencing data, and its application to identify and classify histidine-rich protein 2 and 3 deletions in Plasmodium falciparum
Source: Malar J. 2022 Nov 29;21:357. doi: 10.1186/s12936-022-04376-3 (PMC9706933; doi:10.1186/s12936-022-04376-3)
Supplement: Supplementary file 1 — Additional file 1: Figure S1. Distribution of global isolates including reference strains NF54 (West Africa), 7G8 (Brazil), NF135.C10 (Cambodia), NF166 (Guinea), DD2 (Laos) and HB3 (Honduras). Figure created using Mapchart.net. Figure S2. Subtelomeric read coverage distribution plots. Figure S3. Cambodian sample subset of hrp2/3 and flanking genes. Figure S4. Scatter plots of subtelomeric mean coverage vs. mean coverage of respective HRP-encoding locus. Figure S5. Scatter plots of mean downstream/upstream gene coverage vs. mean coverage of respective HRP-encoding locus. Figure S6. Median standardized coverage by direct sequencing vs sWGA. Figure S7. Proportion of hrp2/3 positions with 0X vs ≥1X coverage for Malawi and Mali samples. Table S1. List of samples that underwent hrp2/3-specific qPCR assay. Table S2. GC3 deletion assignments for hrp2/3 per country. [file 12936_2022_4376_MOESM1_ESM.docx]

**“Gene Coverage Count and Classification” (GC_3_), a locus coverage assessment tool, and its application to identify and classify histidine-rich protein 2 and 3 deletions in *Plasmodium* *falciparum* using short-read whole genome sequencing data**

Thomas C. Stabler*^1, 2^, Ankit Dwivedi^3^, Biraj Shrestha^4^, Sudhaunshu Joshi^4^, Tobias Schindler^1, 2^, Guillermo A. García^5^, Claudia Daubenberger^1, 2^, Joana C. Silva^3, 6^

**Additional file figures/tables**


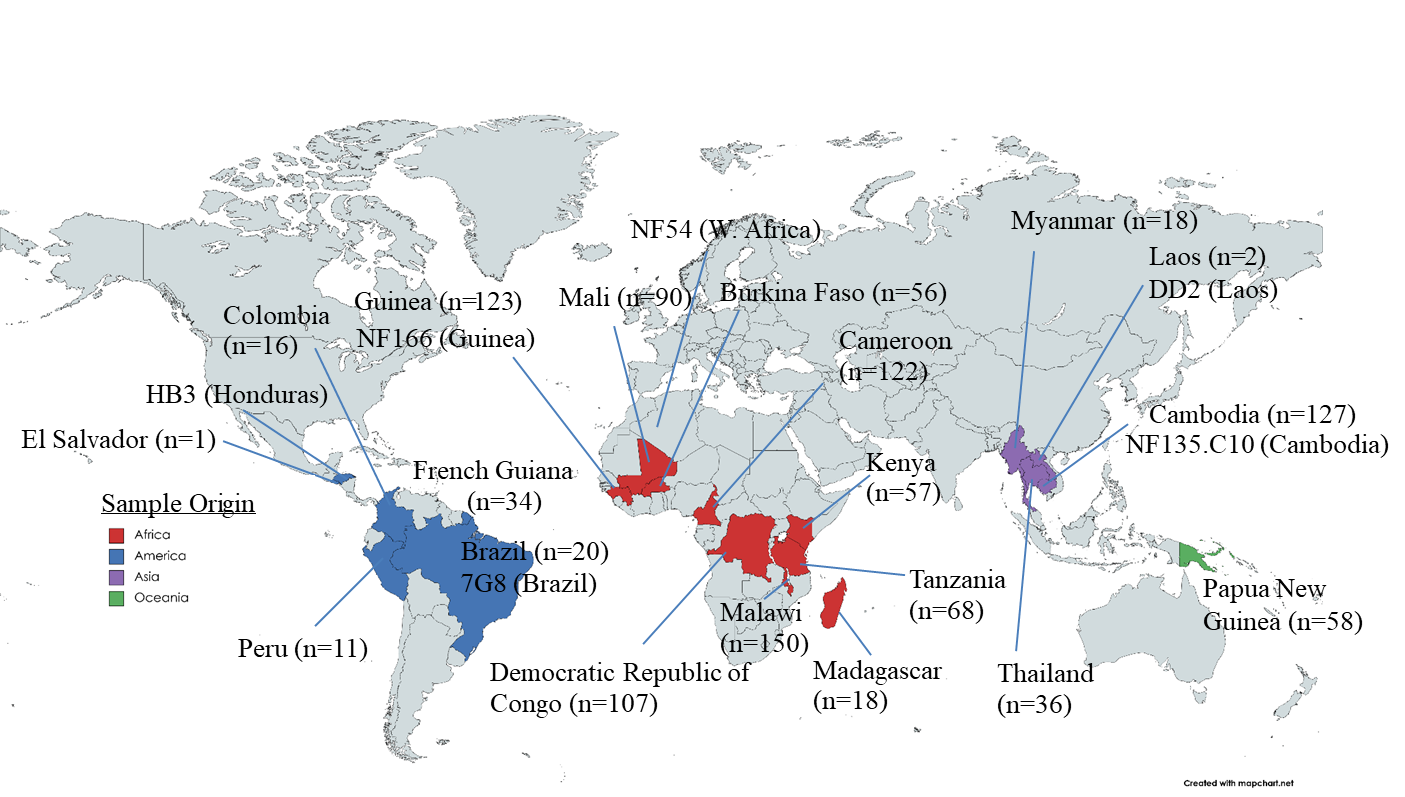


Figure S1. Distribution of global isolates including reference strains NF54 (West Africa), 7G8 (Brazil), NF135.C10 (Cambodia), NF166 (Guinea), DD2 (Laos) and HB3 (Honduras). Figure created using Mapchart.net.


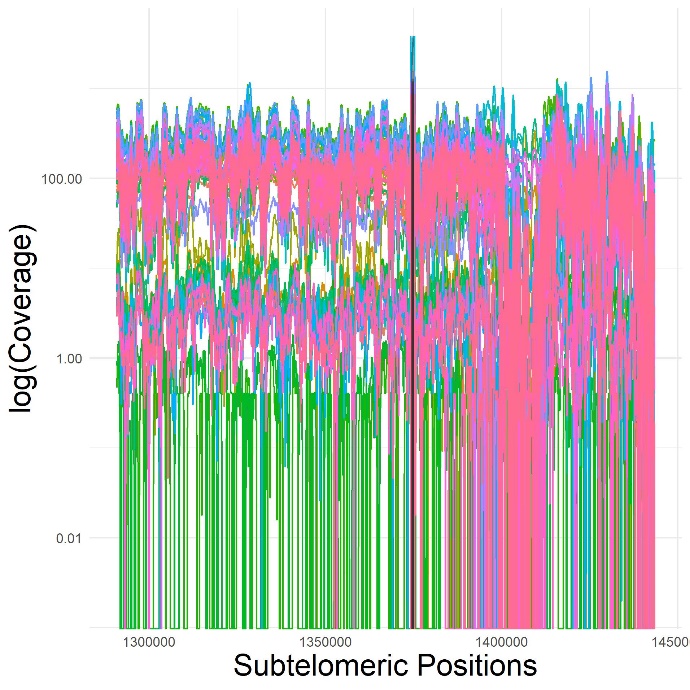

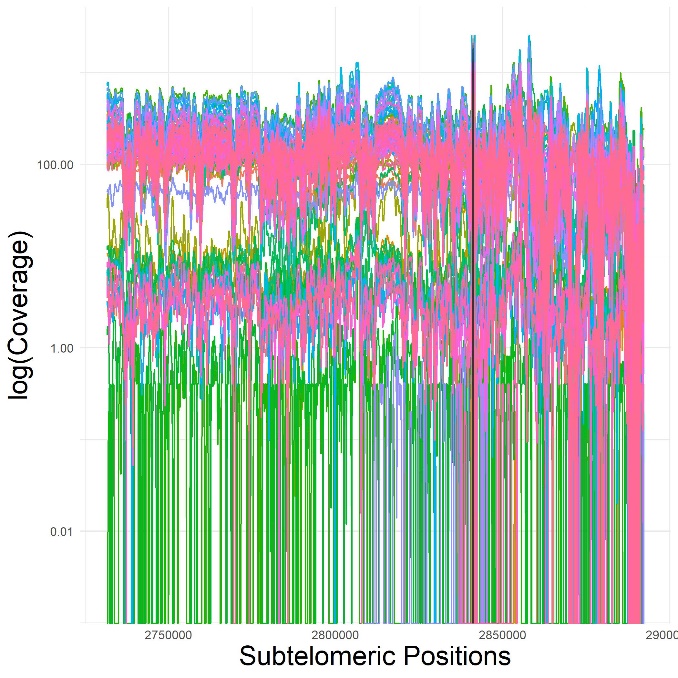


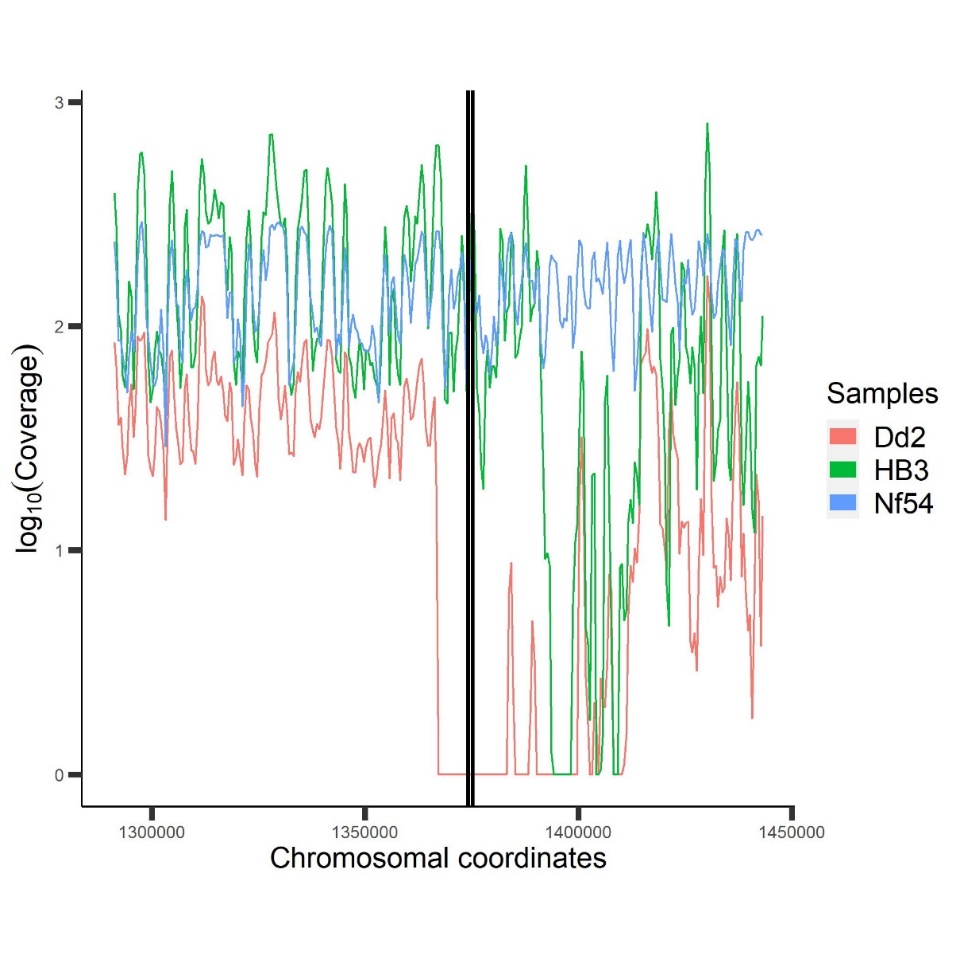

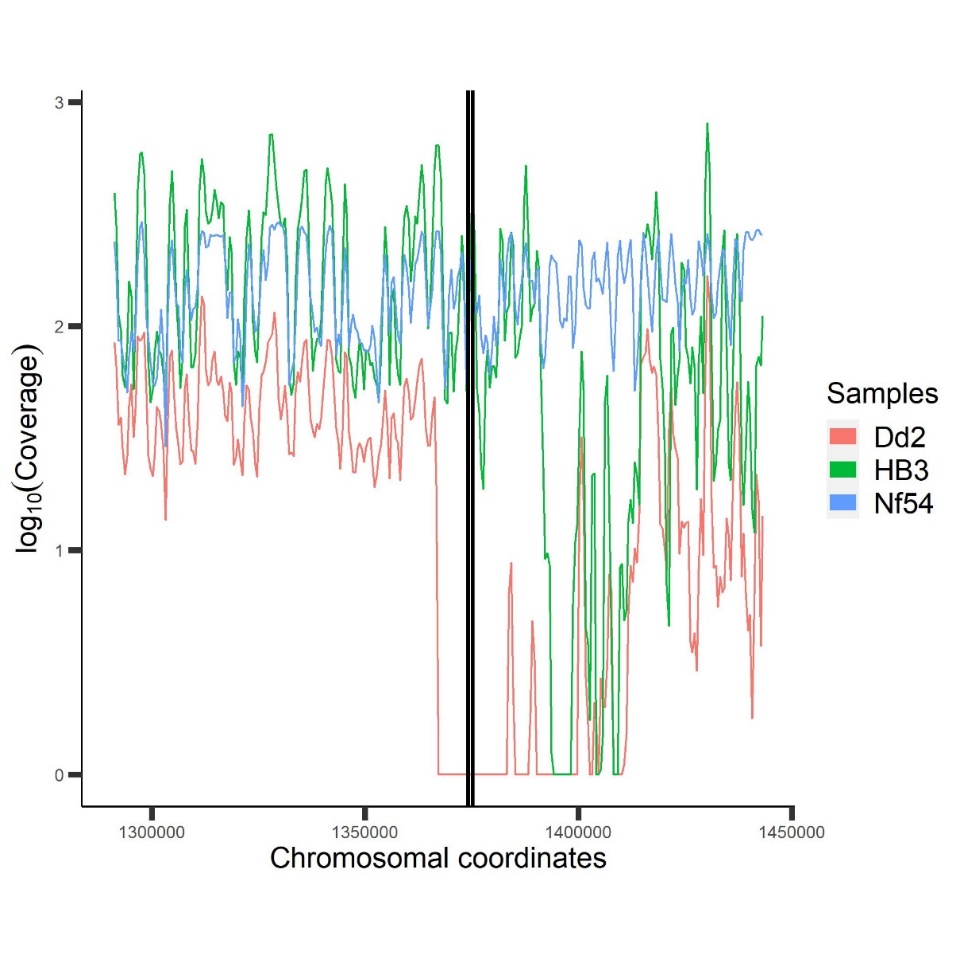

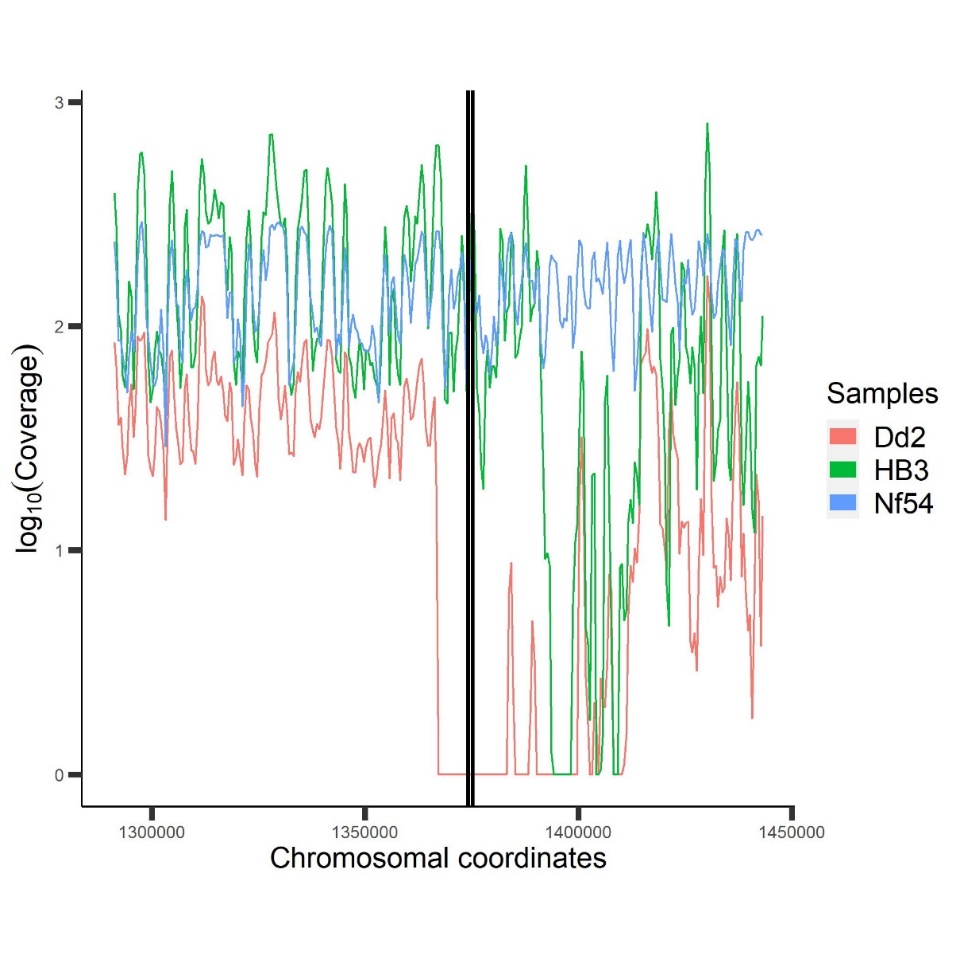

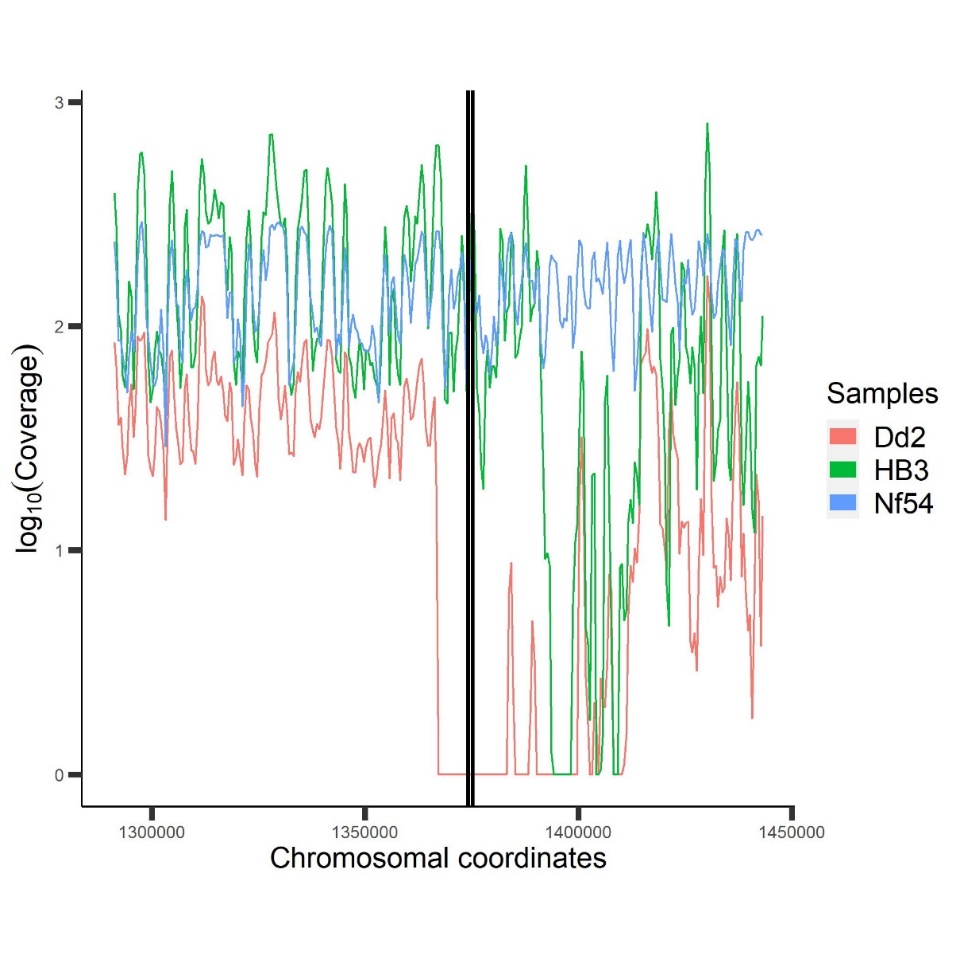


**C
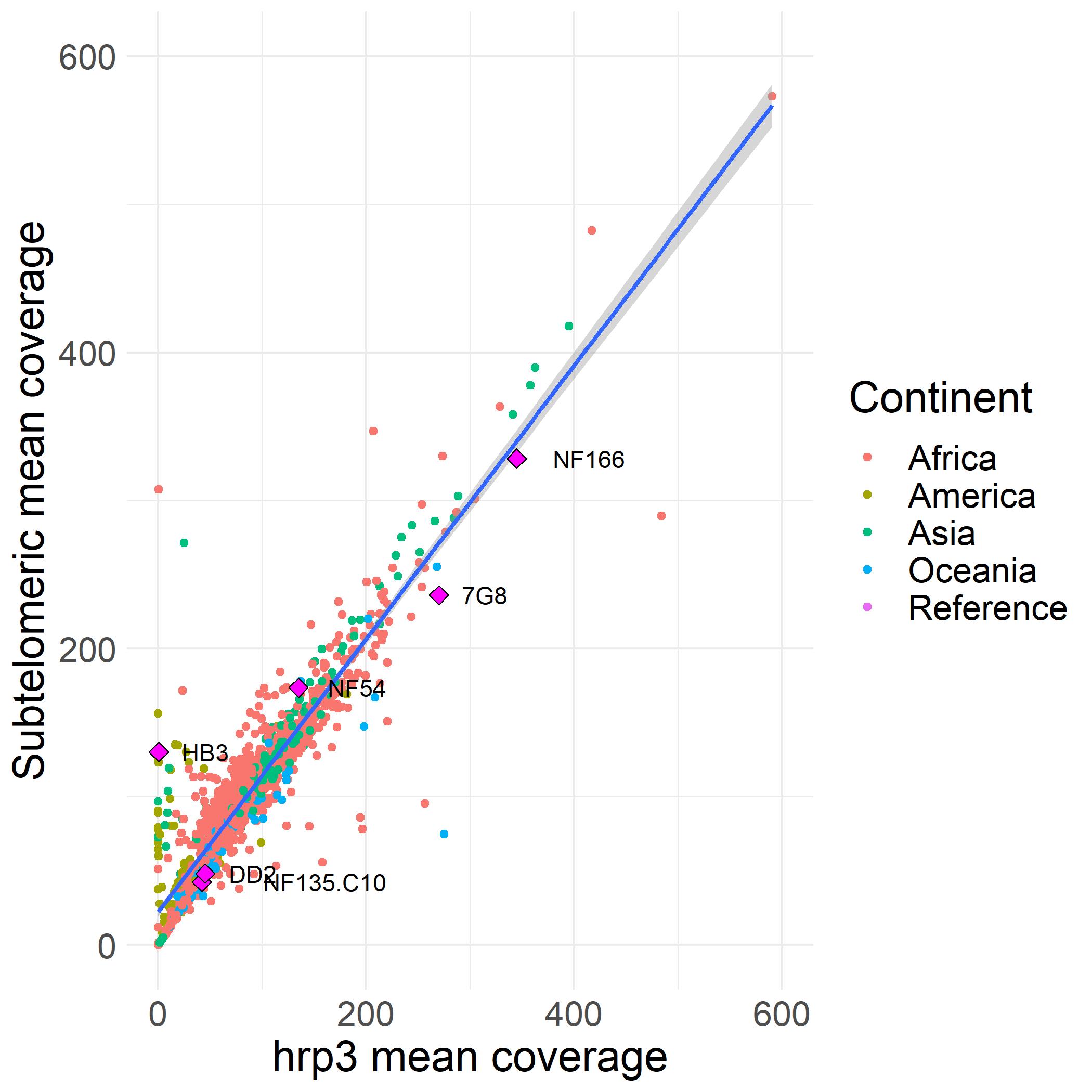
**

**D**

**A
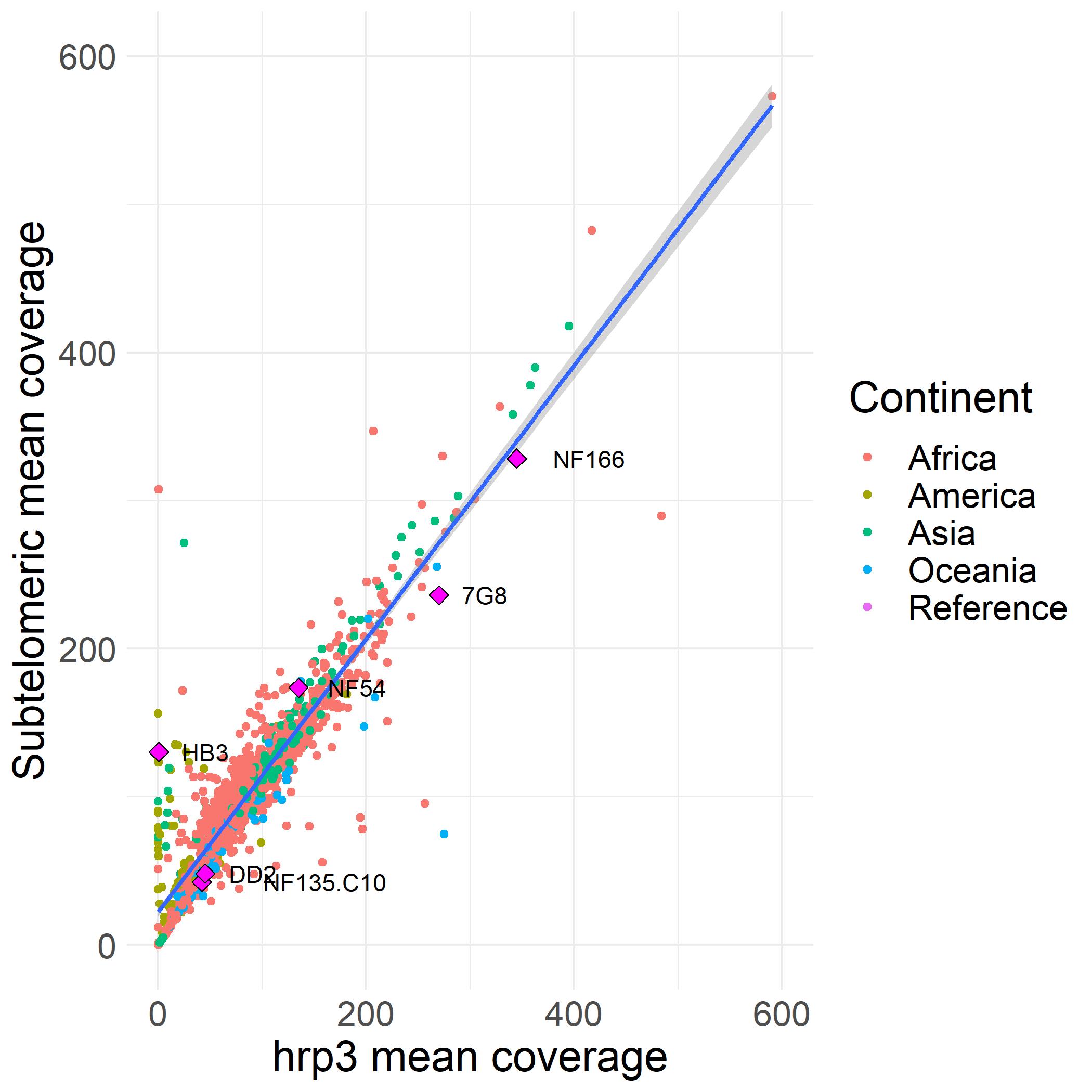
**

**B**


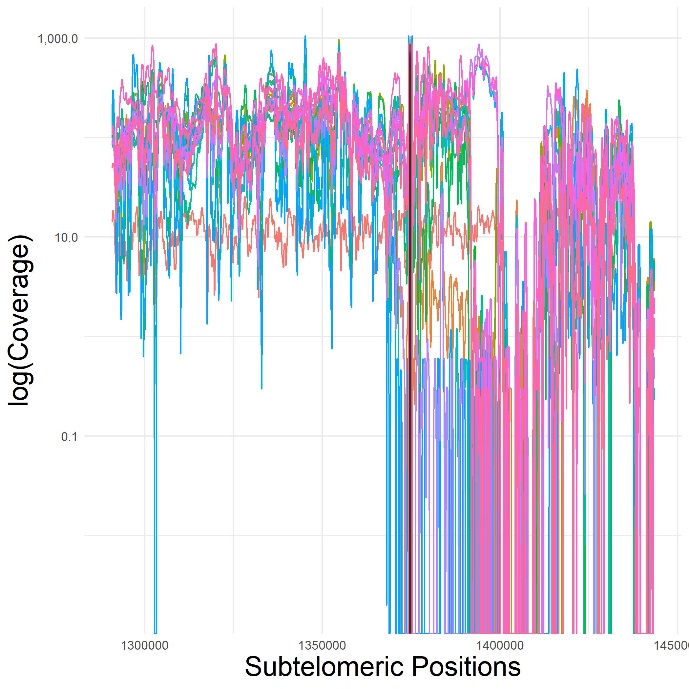

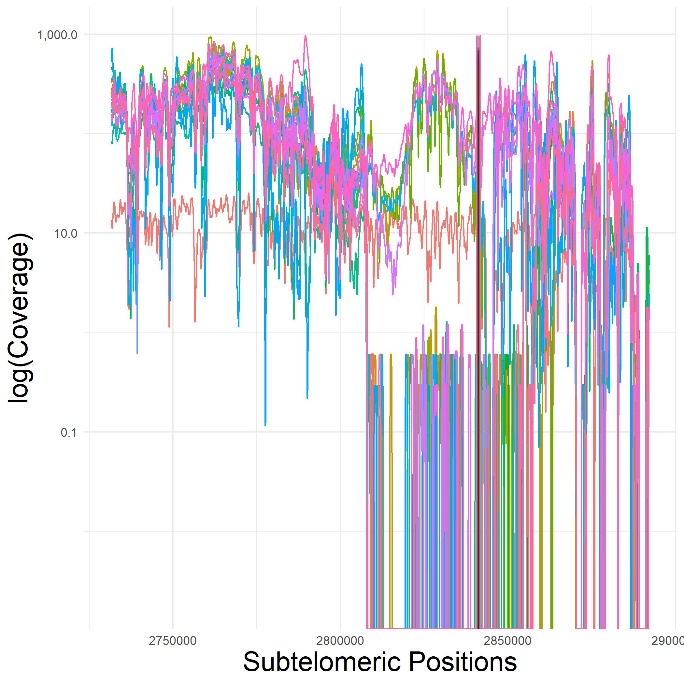


**Figure S2. Subtelomeric read coverage distribution plots.** Log_10_ of subtelomeric coverage among Cambodian (n=128) and Brazilian (n=20) samples where each colored line represents an individual sample (no grouping). Coverage was measured using a 500 bp sliding window with a 100 bp “step” between windows along the genomic segments of interest. **A**. Cambodian samples, chromosome 08; **B.** Cambodian samples, chromosome13; **C.** Brazilian samples, chromosome 08; and **D**. Brazilian samples, chromosome 13. Black line denotes the location of hrp2 (positions 1374236 – 1375299) or hrp3 (positions 2841703 - 2840727). Figures showcase GC_3_’s outputs without grouping, and the type of variability in coverage that can be observed in the subtelomeric region.


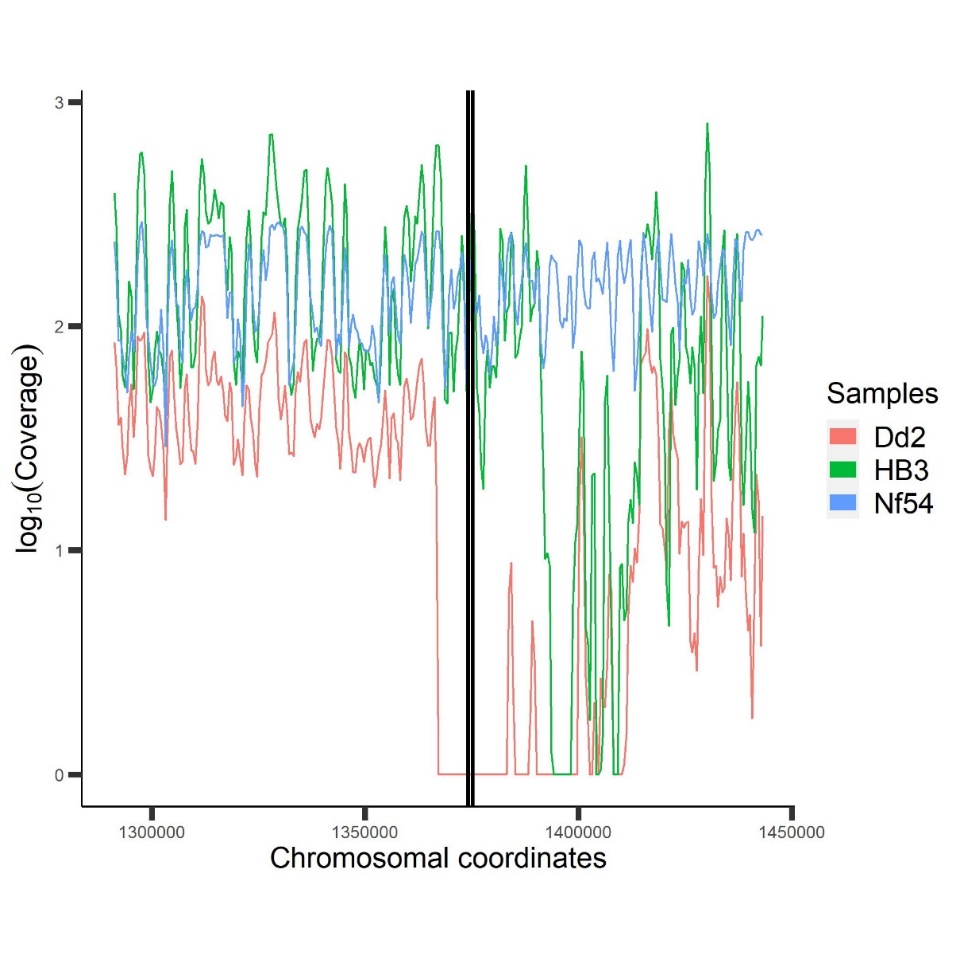

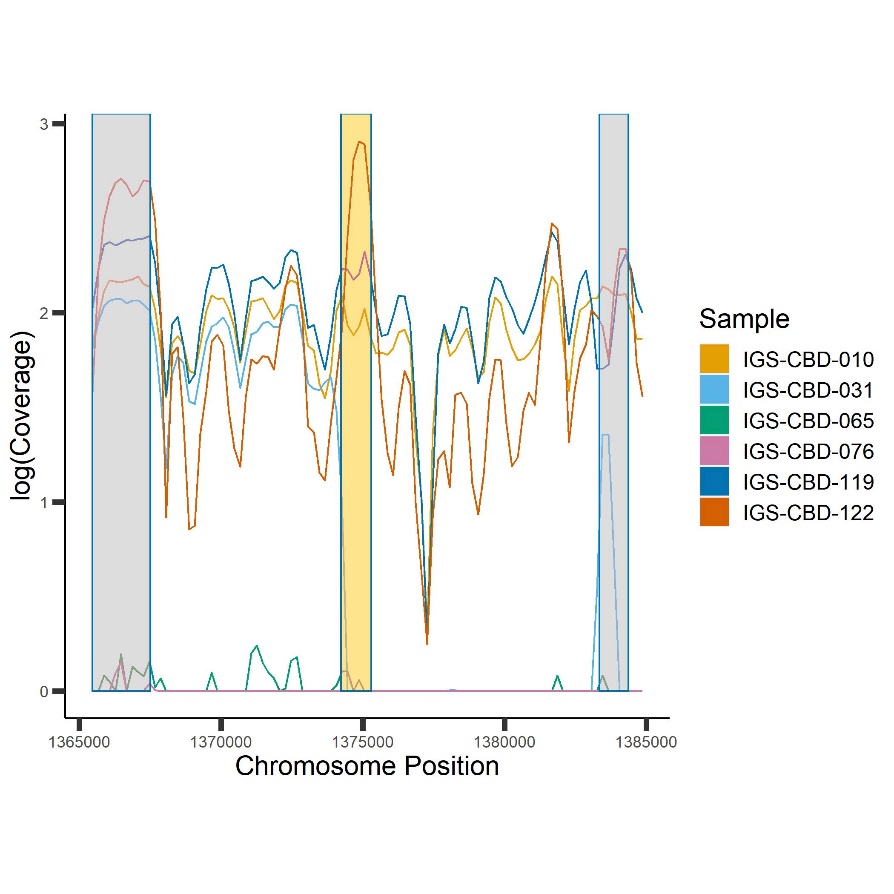


**A
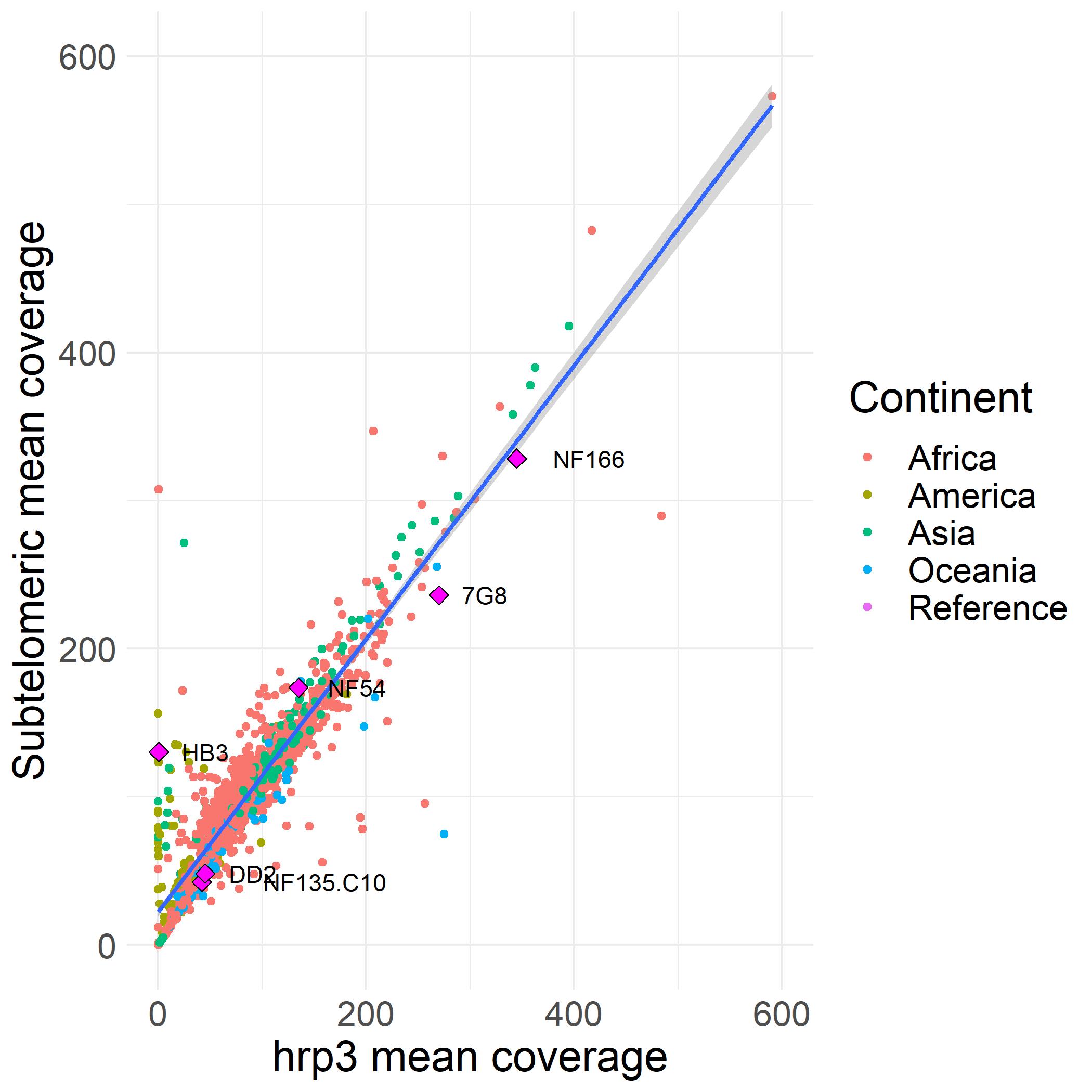
**

**B**


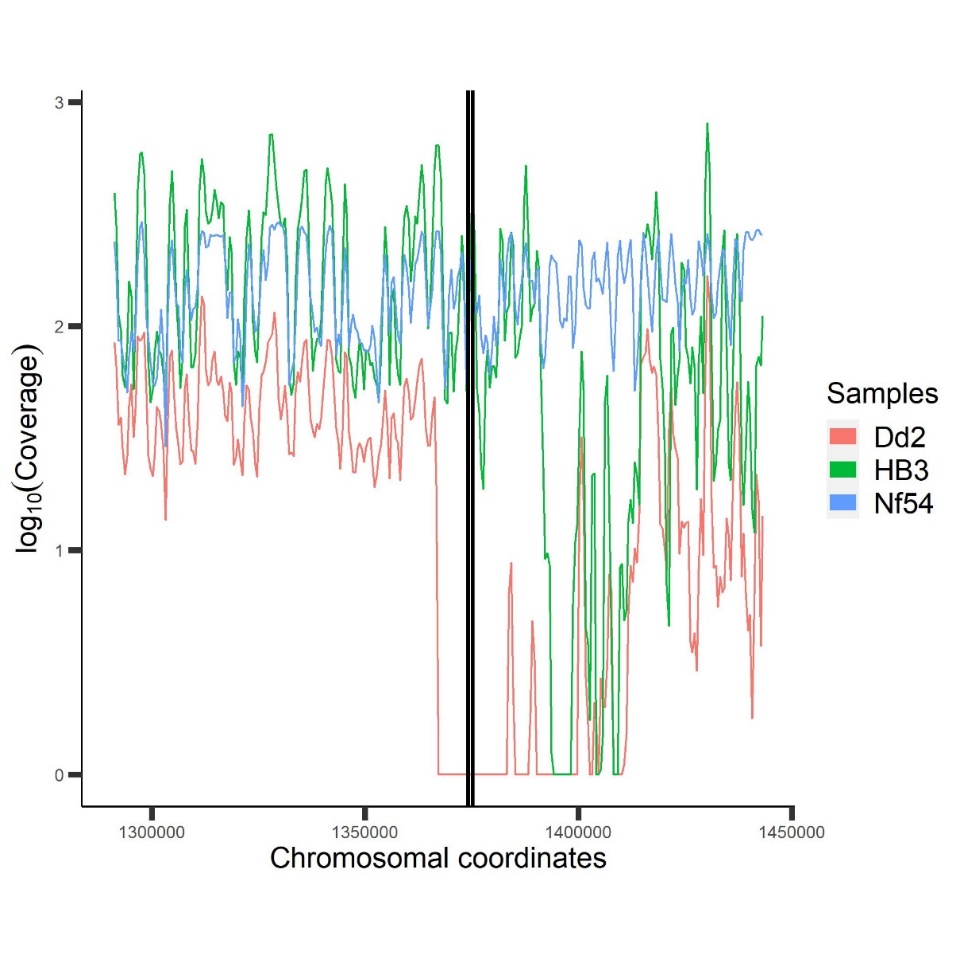

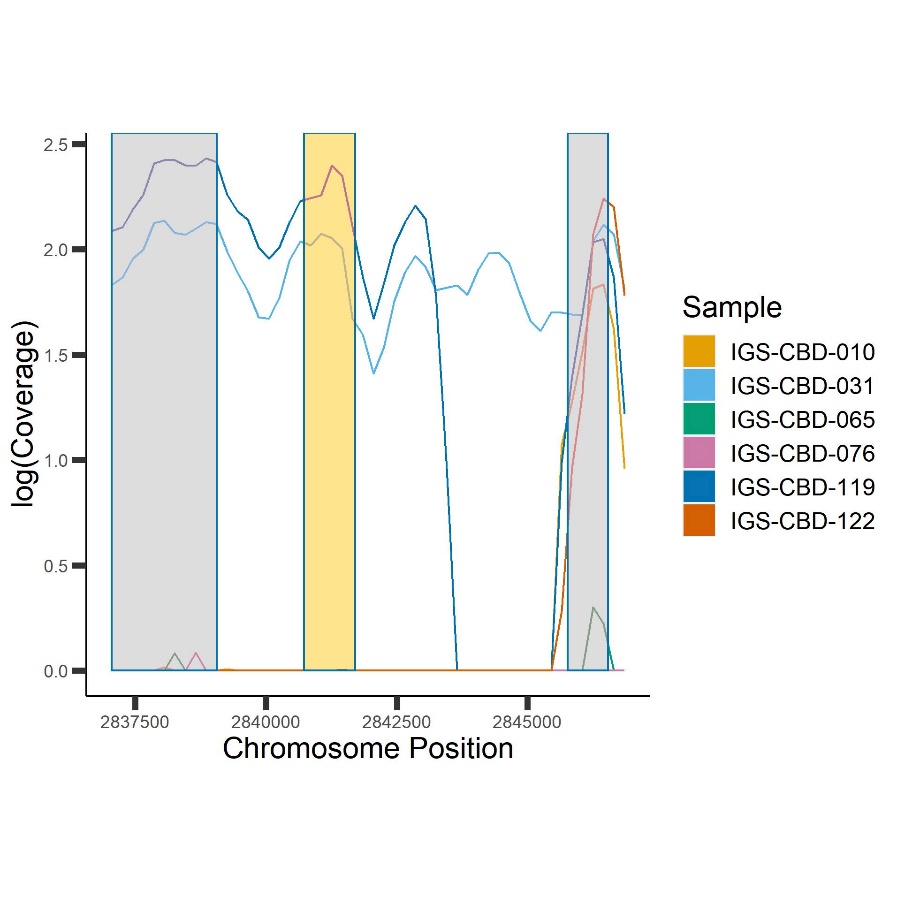


**Figure S3.** **Cambodian sample subset of hrp2/3 and flanking genes**. Log_10_ coverage among Cambodian sample subset (n=6). Negative log_10_ values (<1) were set to 0. Coverage was measured using a sliding window interval of 500bp with a 250 “step” between intervals. **A.** Cambodian sample subset of hrp2 (Positions 1374236 – 1375299) in tan and flanking upstream (Positions 1383349 – 1384377) and downstream (Positions 1365467 – 1367506) genes in grey. **B.** Cambodian sample subset of hrp3 (Positions 2841703 - 2840727) in tan and flanking upstream (Positions 2845767 - 2846538) and downstream (Positions 2837053 - 2839058) genes in grey. Figures suggest a lack of pattern between assigned hrp2/3 deletions and flanking deletions indicative of the larger Cambodian sample set. Among assigned hrp2 deletions (n=3), 1 sample had a deletion extending into the upstream gene region, whereas among samples with intact hrp2 genes (n=3), flanking gene also remained intact. Among assigned hrp3 deletions (n=4), 1 sample had good coverage on both flanking genes, 2 sample had deletions that included both flanking genes and 1 sample had a downstream gene deletion. Among samples with good hrp3 coverage (n=2), 1 had intact flanking genes and 1 reported a downstream flanking gene deletion.


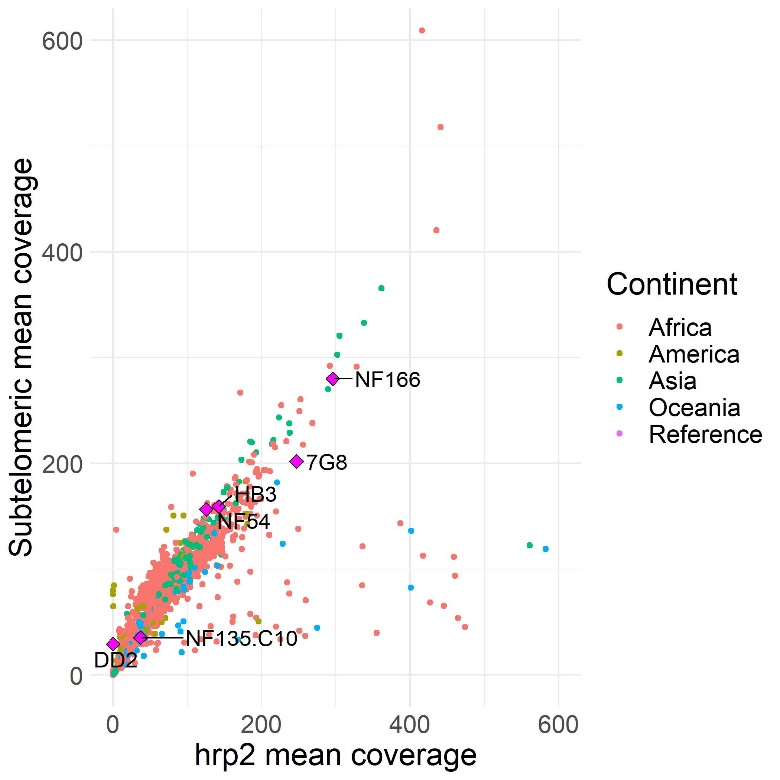

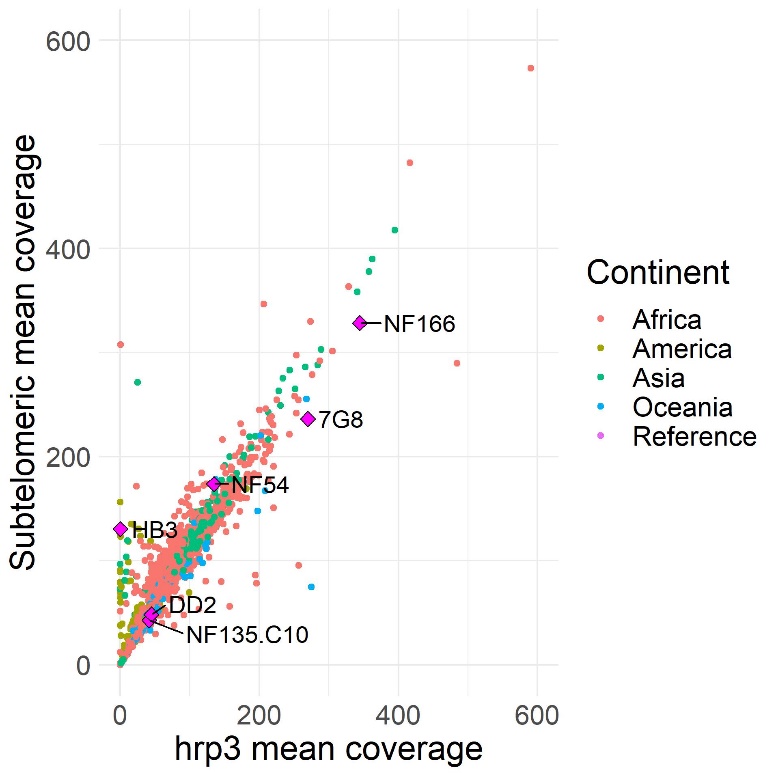


R-squared = 0.90

R-squared = 0.88

**A
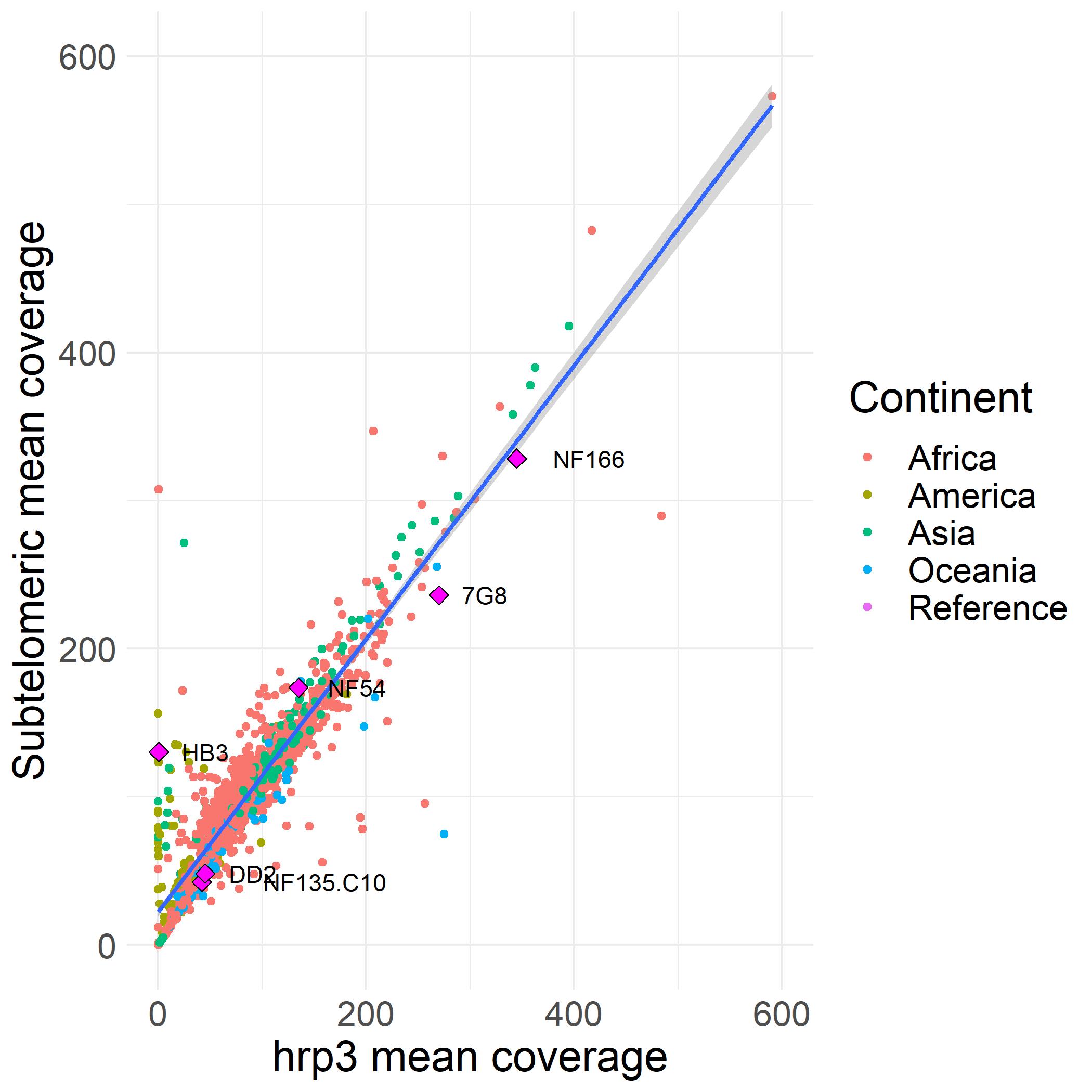
**

**B**

**Figure S4. Scatter plots of subtelomeric mean coverage vs. mean coverage of respective HRP-encoding locus.**  **A.** chromosome 8 compared to hrp2 mean coverage (eight outlier points not shown) and **B.** subtelomeric mean coverage of chromosome 13 compared to hrp3 mean coverage (two outlier points not shown). A total of 1,329 global isolates from 22 different countries were examined, including six reference strains of known coverage. Colors represent samples by continent and reference strains; reference strains are additionally labeled with their respective name. Spearman’s rank correlation coefficient was used to measure relationship between parameters and R-squared values are reported. Analysis and figures were generated using R v4.1.0. The majority of coverage on the hrp2/3 locus is similar to the coverage of the respective subtelomeric region, however some African samples show higher hrp2 coverage than the corresponding subtelomeric region. Additionally, a cluster of American and Asian samples has very little hrp3 coverage compared to the respective subtelomeric region, suggestive of the higher frequency of hrp3 deletions in these regions.


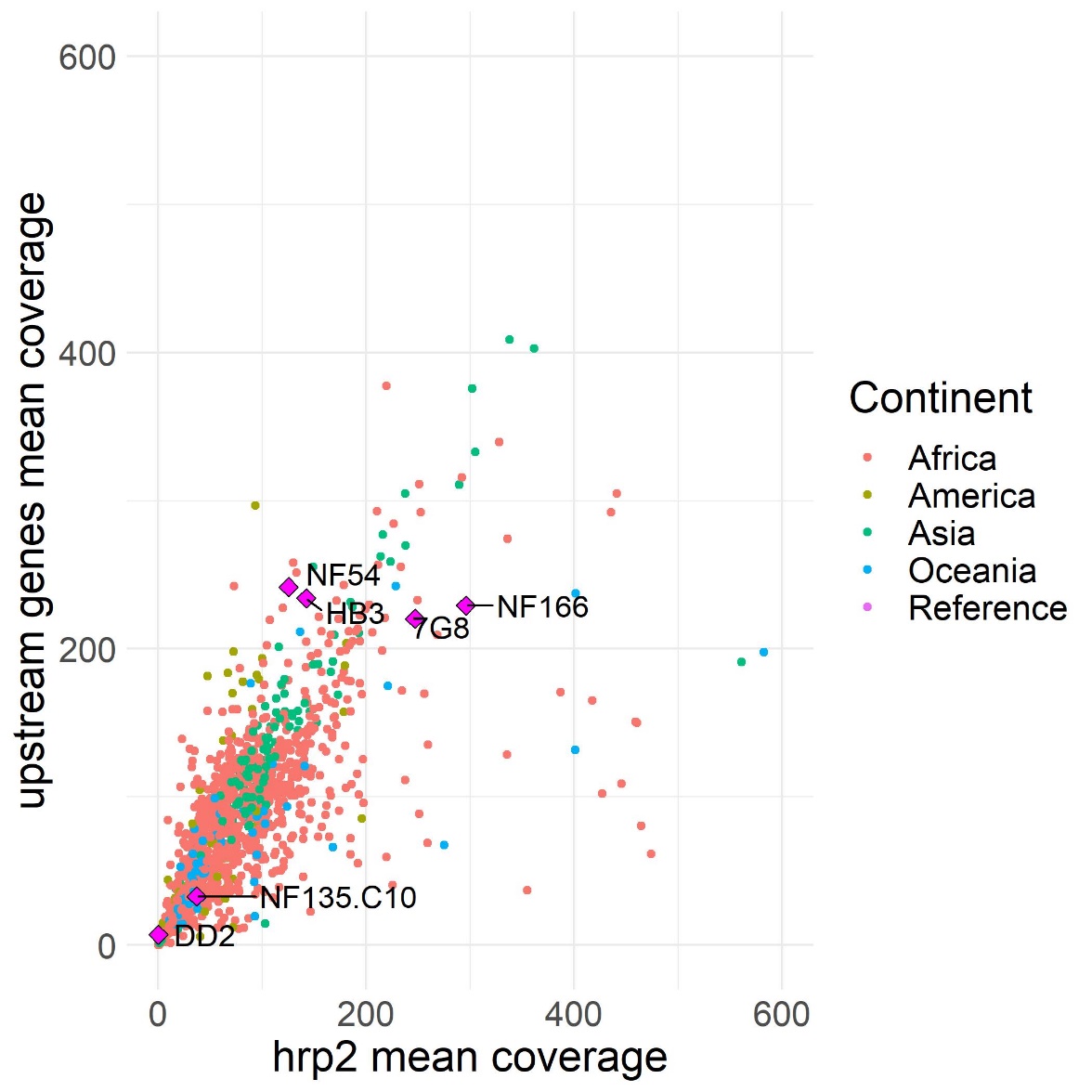

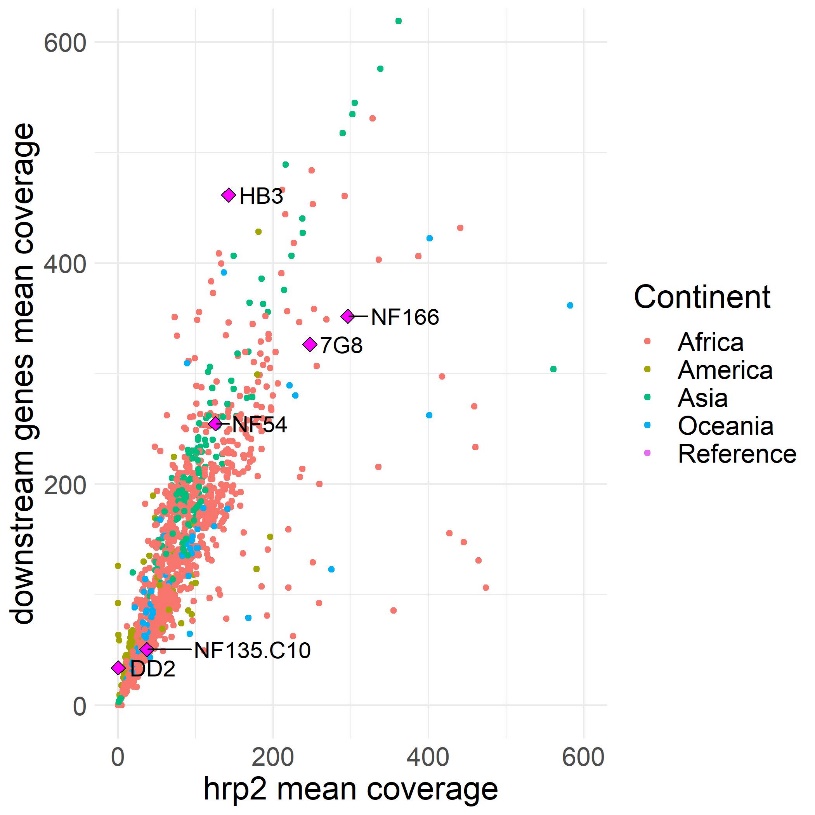

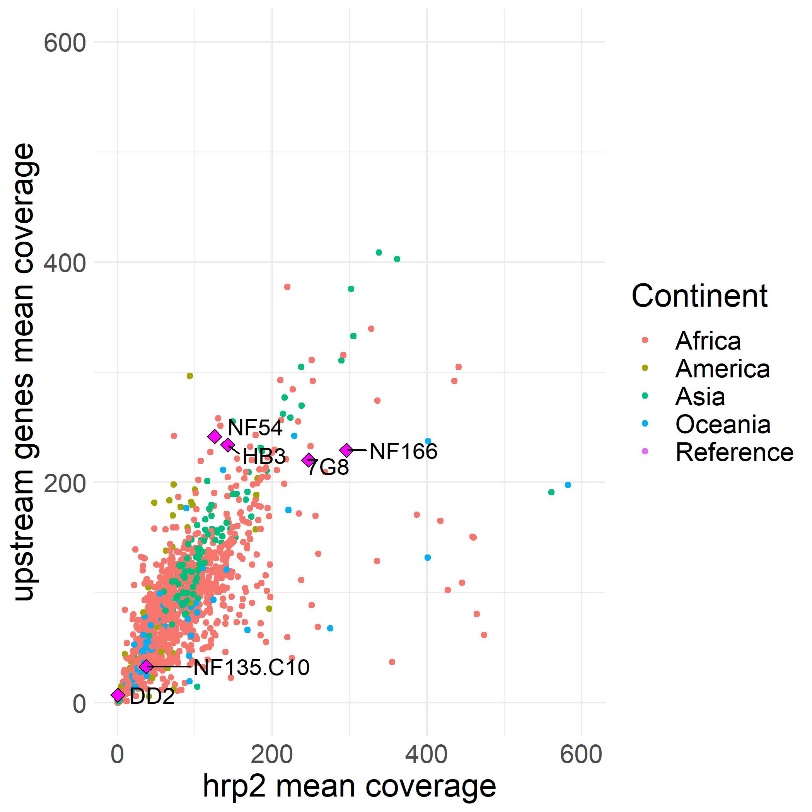


R-squared = 0.81

R-squared = 0.88

**C
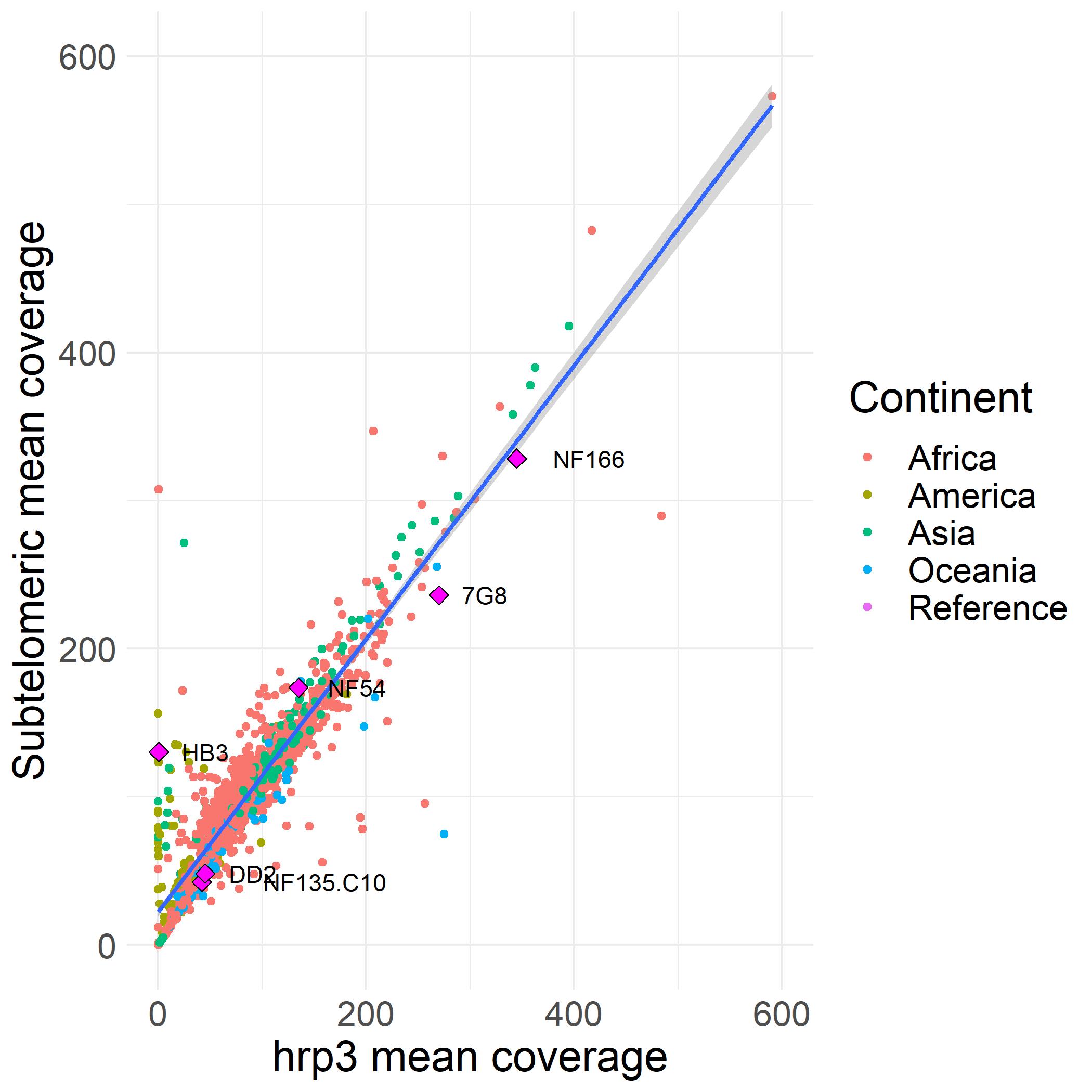
**

**D**

**A
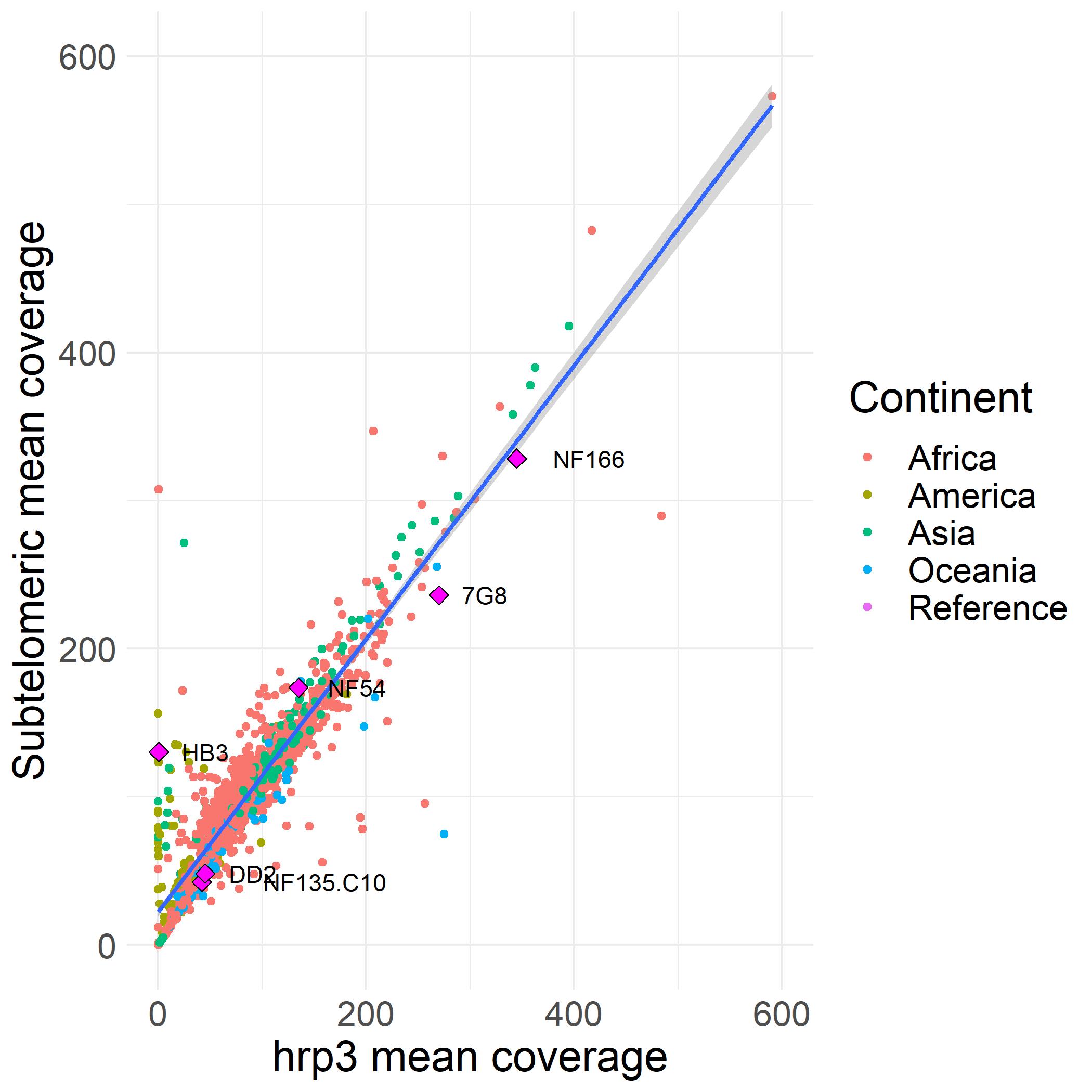
**

**B**


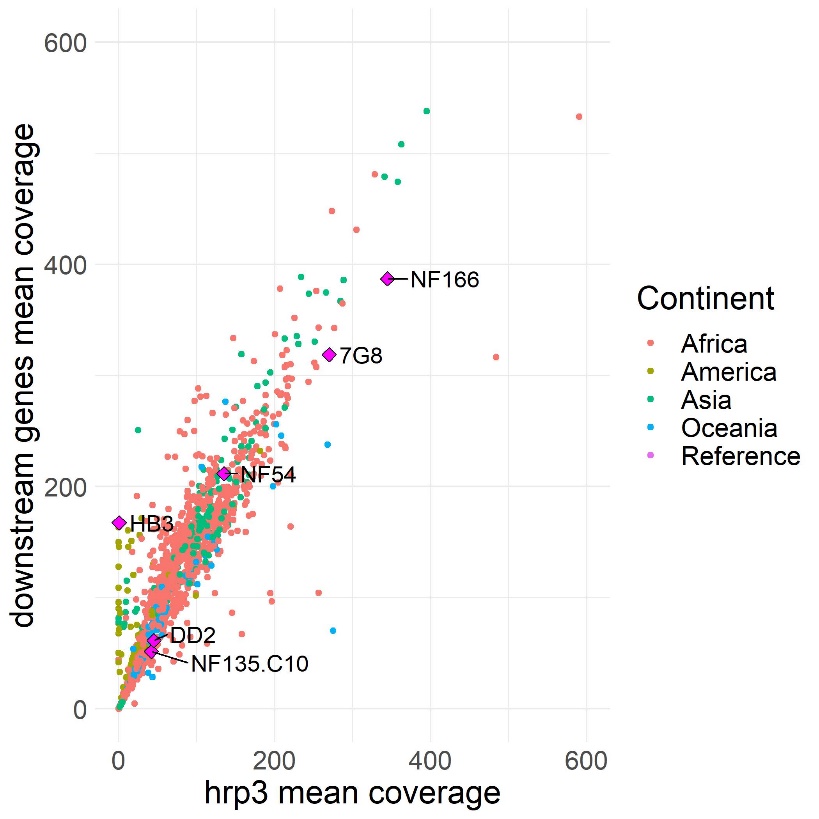

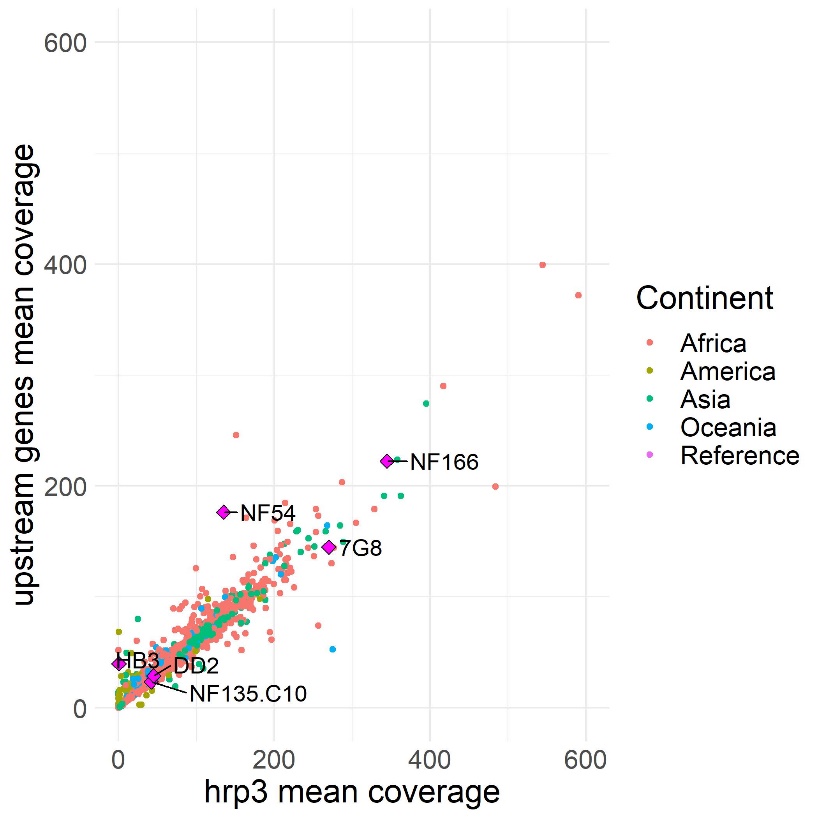


R-squared = 0.76

R-squared = 0.84

**Figure S5. Scatter plots of mean downstream/upstream gene coverage vs. mean coverage of respective HRP-encoding locus. A.** hrp2 mean coverage compared to mean coverage of downstream gene (10 outlier points not shown). Downstream flanking genes included PHISTa pseudogene, heat shock protein 70, and the sporozoite invasion-associated protein 2. **B.** hrp2 mean coverage compared to upstream gene’s mean coverage (9 outlier points not shown). Upstream flanking genes included PHIST pseudogene, Stevor and PfEMP. **C.** hrp3 mean coverage compared to downstream gene’s mean coverage (2 outlier points not shown). Downstream flanking genes included PHISTb of unknown function and DNA repair endonuclease XPF protein. **D.** hrp3 mean coverage compared to upstream gene’s mean coverage (all points shown). Upstream flanking genes included PHIST of unknown function and PfEMP1. A total of 1,329 global isolates from 22 different countries were examined along with 6 reference strains of known coverage. Spearman’s rank correlation coefficient were calculated to measure relationship between parameters and is reported as R-squared values. Analysis and figures were generated using R v4.1.0. In general, mean hrp2/3 mean coverage is slightly lower to mean coverage of mean downstream gene coverage, possibly due to downstream genes closer chromosomal position to the core genome where coverage is typically improved.

**A
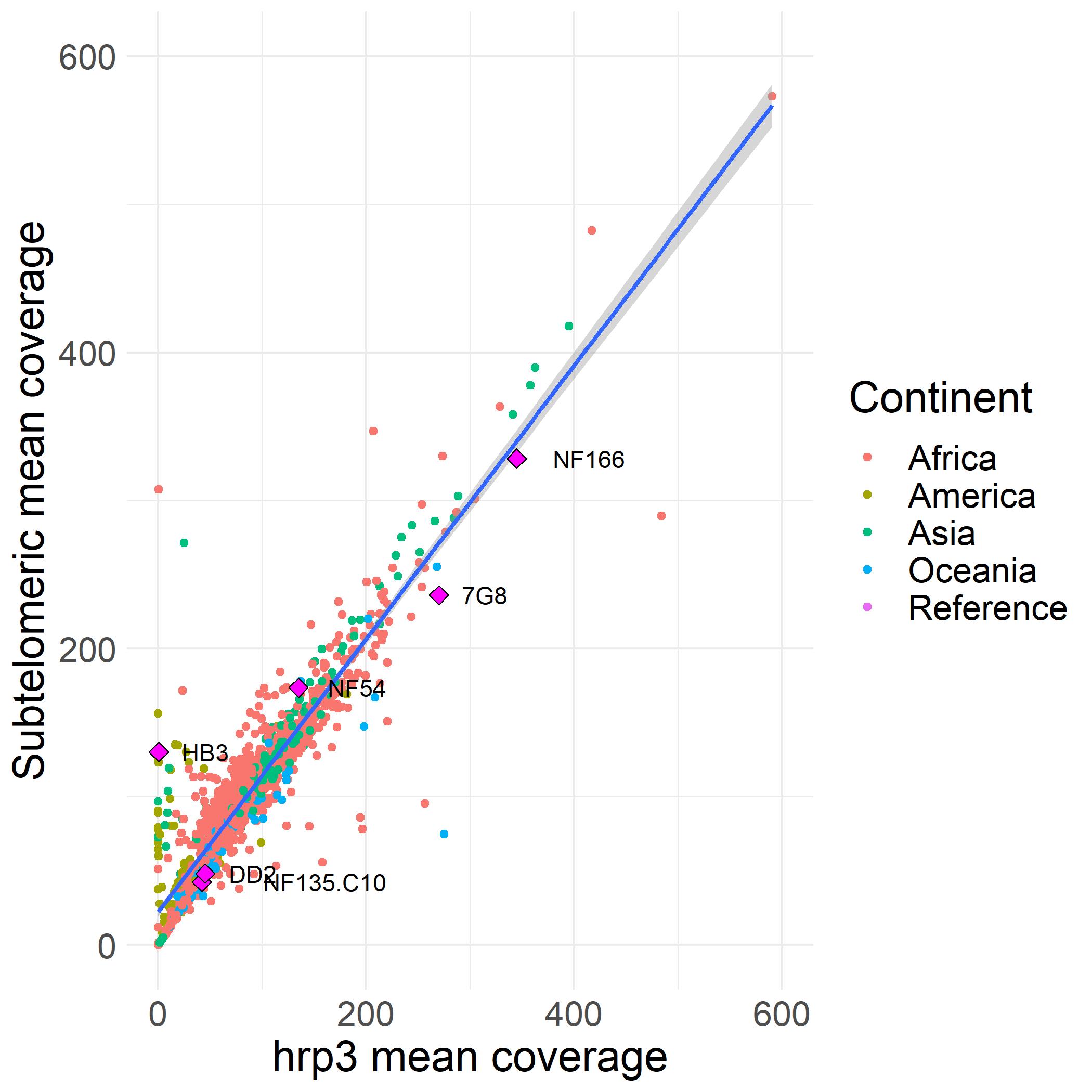
**

**B**


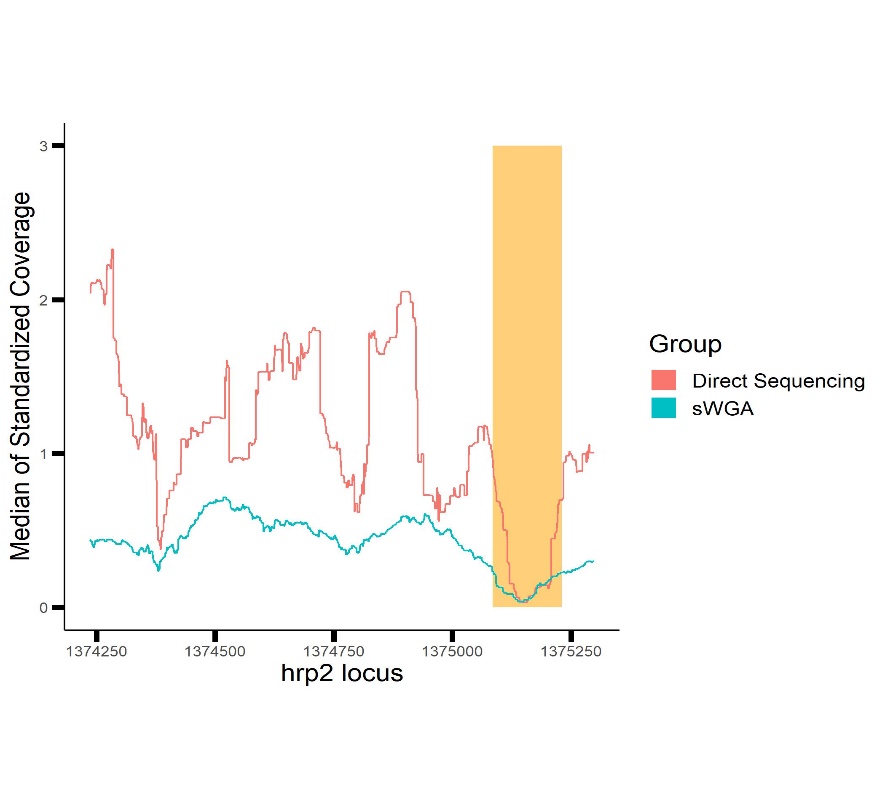

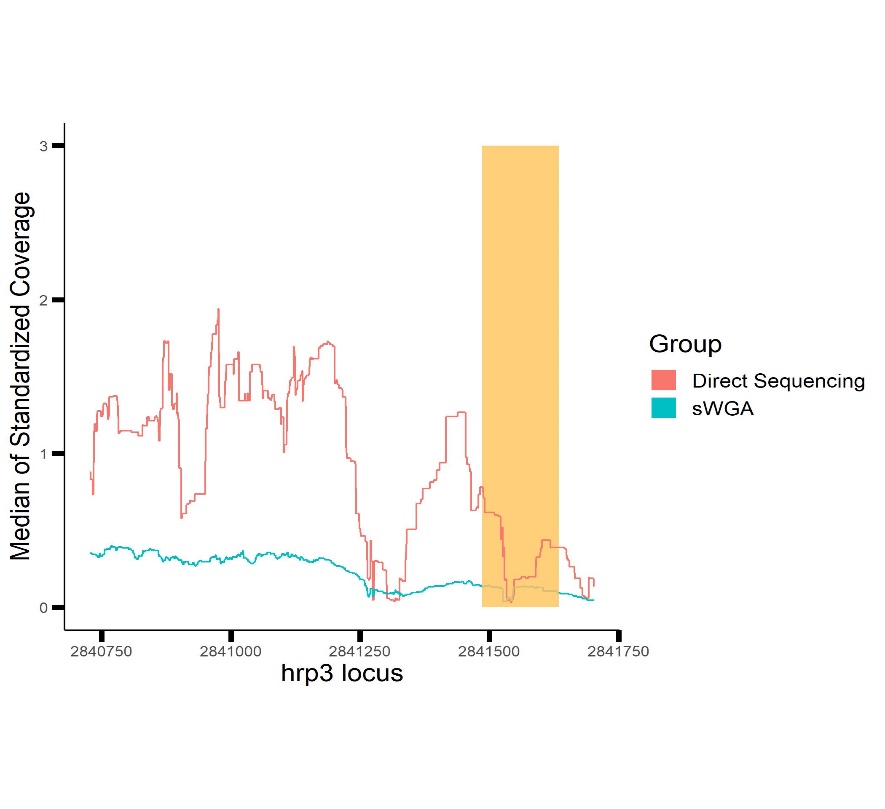


**Figure S6. Median standardized coverage by direct sequencing vs sWGA.** Median of standardized coverage [SUM (Coverage/Subtelomeric Mean Coverage)/ Total samples] grouped by duplicate samples directly sequenced vs. selective whole genome amplification (sWGA) prior to sequencing. Samples were plotted along **A.** hrp2 positions on chromosome 8 and **B.** hrp3 positions on chromosome 13. Tan shading marks intron positions of target gene, whereas unshaded areas are exon positions (hrp2 -> exon 1: 1375299 – 1385231; intron: 1375230 – 1375085; exon 2: 1375084 – 1374236; hrp3 -> exon 1: 2841703 – 2841635; intron: 2841634 – 2841486; exon 2: 2841485 – 2840727). Analysis and figures were generated using R v4.1.0. When samples undergo sWGA prior to sequencing, mean subtelomeric coverage appears to increase, but not necessarily at the hrp2/3 positions, resulting in lower standardized coverage values.

**Figure S7. Proportion of hrp2/3 positions with 0X vs ≥1X coverage for Malawi and Mali samples.** Proportional sample count of hrp2 and hrp3 gene positions with zero coverage vs. positions with ≥1X coverage among **A.** Malawian samples (n=150) and **B.** Malian samples (n=90). Tan section on each plot represents the intron region of each respective gene. Analysis and figures were generated using R v4.1.0. Malawi hrp2/3 positions had a higher proportion of 0X coverage than Mali, but both sample sets appear to have a low proportion of hrp2/3 positions with 0X coverage.


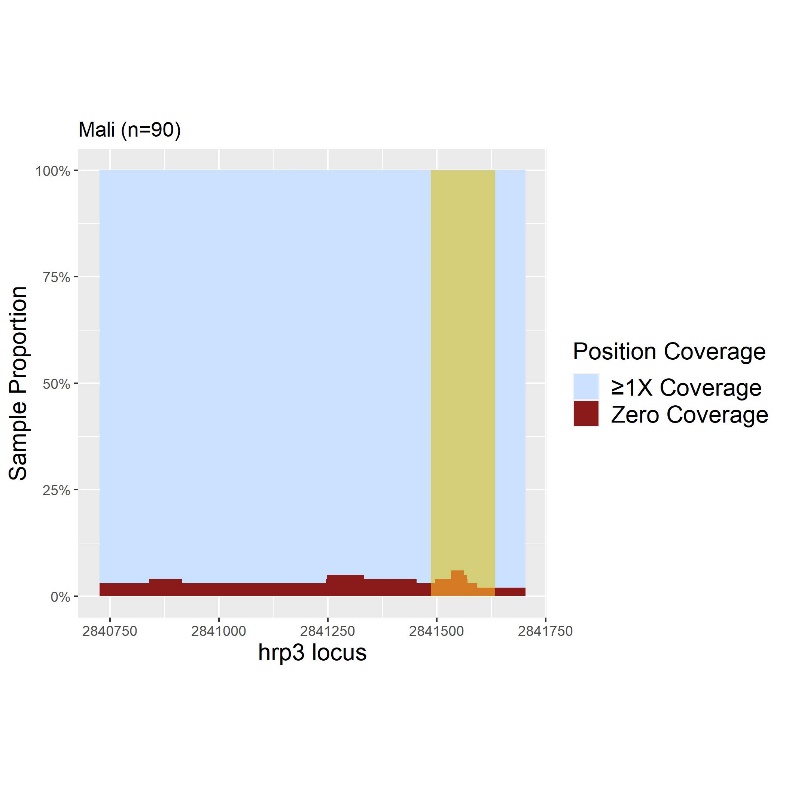

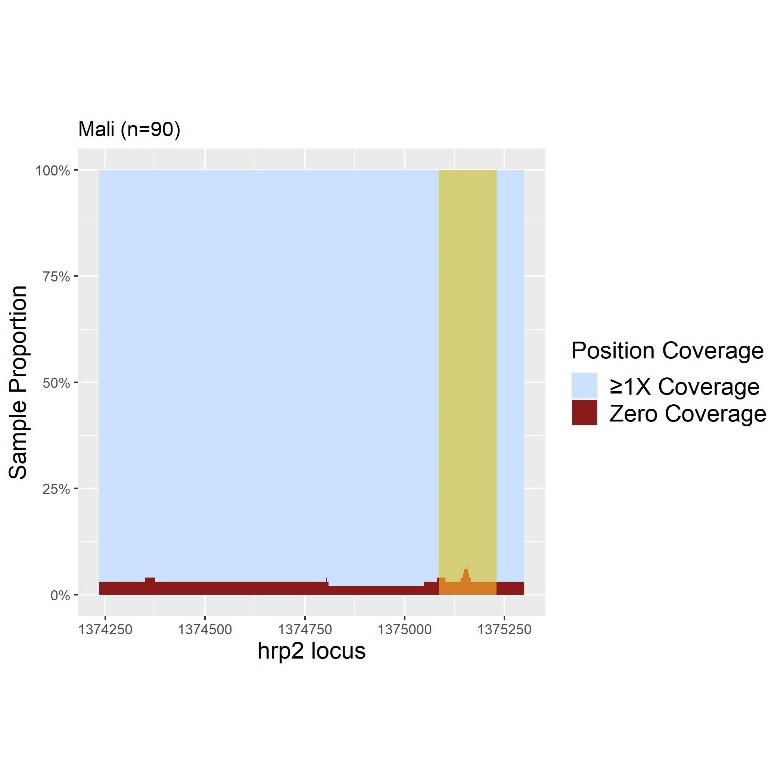

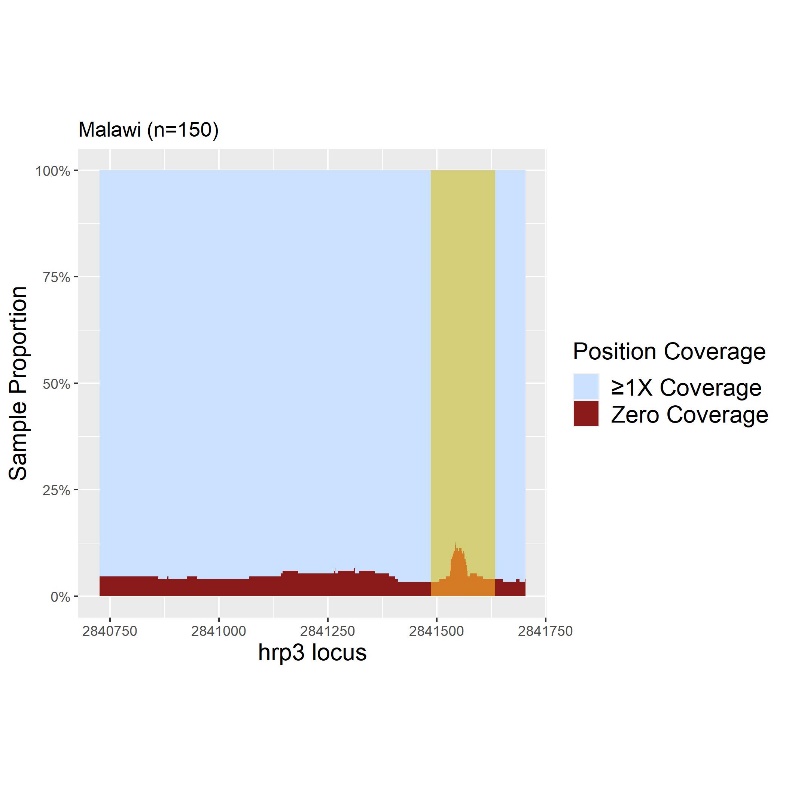

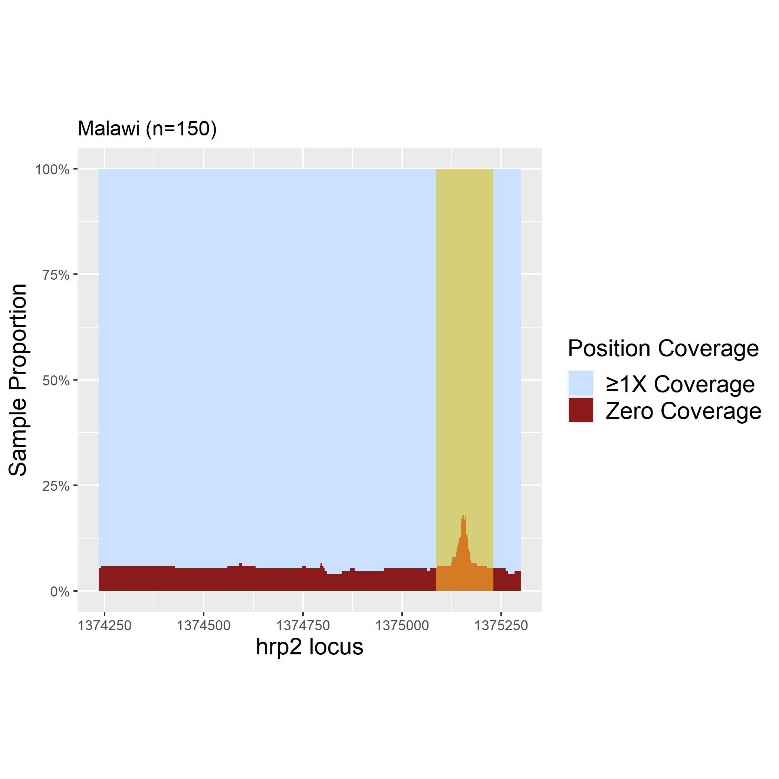


**B**

**Mali**

**A**

**Malawi**

**Table S1. List of samples that underwent hrp2/3-specific qPCR assay.** List of global samples that underwent hrp2/3-specific qPCR assay(Schindler T., et al. 2019). Samples were subgrouped bassed specific GC_3_ results of interest then randomly selected using R v4.1.0. Two samples were excluded due to low parasitemia resulting in low or no detection of qPCR control gene (Cq cutoff >37.5).

| **Sample ID** | **Country of Collection** | **Source of Sequencing** | **SRA Accession** | **Subgroup** | **Comment(s)** |
| --- | --- | --- | --- | --- | --- |
| IGS-BRA-001sA | Brazil | IGS | SAMN08815883 | double hrp2/3 deletion |  |
| IGS-BRA-017sA | Brazil | IGS | SAMN08815899 | No Deletions |  |
| IGS-BRA-021 | Brazil | IGS | SAMN08815903 | No Deletions |  |
| IGS-CBD-008 | Cambodia | IGS | SAMN06175894 | Low gene coverage |  |
| IGS-CBD-026 | Cambodia | IGS | SAMN06175913 | No Deletions |  |
| IGS-CBD-031 | Cambodia | IGS | SAMN06175918 | hrp2 deletion (complete) |  |
| IGS-CBD-034 | Cambodia | IGS | SAMN06175921 | hrp2 PCR primer deletion |  |
| IGS-CBD-094 | Cambodia | IGS | SAMN06175982 | hrp3 PCR primer deletion |  |
| IGS-CBD-099 | Cambodia | IGS | SAMN06175987 | hrp3 PCR primer deletion |  |
| IGS-MLI-031 | Mali | IGS | SAMN06175812 | hrp3 discordant pair |  |
| IGS-MLI-036 | Mali | IGS | SAMN06175817 | hrp3 deletion (complete) |  |
| IGS-MLI-039 | Mali | IGS | SAMN06175820 | hrp2 discordant pair |  |
| IGS-MWI-016sA | Malawi | IGS | SAMN08815781 | hrp2 PCR primer deletion | Excluded |
| IGS-MWI-077sA | Malawi | IGS | SAMN08815842 | double hrp2/3 deletion | Excluded |
| IGS-MWI-251sA | Malawi | IGS | SAMN08849517 | Low gene coverage |  |
| IGS-MWI-254sA | Malawi | IGS | SAMN08849521 | Low gene coverage |  |
| IGS-THL-017 | Thailand | IGS | SAMN06175868 | No Deletions |  |

**Table S2. GC_3_ deletion assignments for hrp2/3 per country.** Global isolates (n=1,114) were assigned hrp2/3 deletions to samples with >25% zero coverage positions on coding region (exon1 and exon2). Of note is the increased proportion of hrp3 deletions among Brazil and Cambodia sample sets.

| Continent | Country | n | hrp2/hrp3 genotype (n)* | | | |
| --- | --- | --- | --- | --- | --- | --- |
|  |  |  | +/+ | -/+ | +/- | -/- |
| Africa | Burkina Faso | 56 | 56 | 0 | 0 | 0 |
|  | Cameroon | 122 | 122 | 0 | 0 | 0 |
|  | Dominican Republic of Congo (DRC) | 107 | 107 | 0 | 0 | 0 |
|  | Guinea | 123 | 123 | 0 | 0 | 0 |
|  | Kenya | 57 | 56 | 0 | 1 | 0 |
|  | Madagascar | 18 | 18 | 0 | 0 | 0 |
|  | Malawi | 150 | 141 | 0 | 0 | 9 |
|  | Mali | 90 | 87 | 0 | 0 | 3 |
|  | Tanzania | 68 | 68 | 0 | 0 | 0 |
| **Total** | | **791** | **778** | **0** | **1** | **12** |
| America | Brazil | 20 | 5 | 0 | 11 | 4 |
|  | Colombia | 16 | 16 | 0 | 0 | 0 |
|  | El Salvador | 1 | 1 | 0 | 0 | 0 |
|  | French Guiana | 34 | 32 | 0 | 1 | 1 |
|  | Peru | 11 | 8 | 1 | 2 | 0 |
| **Total** | | **82** | **62** | **1** | **14** | **5** |
| Asia | Cambodia | 127 | 116 | 1 | 8 | 2 |
|  | Laos | 2 | 2 | 0 | 0 | 0 |
|  | Myanmar | 18 | 18 | 0 | 0 | 0 |
|  | Thailand | 36 | 35 | 0 | 0 | 1 |
| **Total** | | **183** | **171** | **1** | **8** | **3** |
| Oceania | Papua New Guinea (PNG) | 58 | 58 | 0 | 0 | 0 |
|  |  |  |  |  |  |  |
| **Total** | | **1114** | **1069** | **2** | **23** | **20** |
| *deletions assigned to isolates with >25% zero coverage positions on the *hrp2/3* coding region | | | | | | |

See additional File 3 for Tables S3 – S5
